# DNA Is an Antimicrobial Component of Neutrophil Extracellular Traps

**DOI:** 10.1371/journal.ppat.1004593

**Published:** 2015-01-15

**Authors:** Tyler W.R. Halverson, Mike Wilton, Karen K. H. Poon, Björn Petri, Shawn Lewenza

**Affiliations:** University of Calgary, Snyder Institute for Chronic Diseases, Department of Microbiology, Immunology and Infectious Diseases, Calgary, Alberta, Canada; Emory University School of Medicine, UNITED STATES

## Abstract

Neutrophil extracellular traps (NETs) comprise an ejected lattice of chromatin enmeshed with granular and nuclear proteins that are capable of capturing and killing microbial invaders. Although widely employed to combat infection, the antimicrobial mechanism of NETs remains enigmatic. Efforts to elucidate the bactericidal component of NETs have focused on the role of NET-bound proteins including histones, calprotectin and cathepsin G protease; however, exogenous and microbial derived deoxyribonuclease (DNase) remains the most potent inhibitor of NET function. DNA possesses a rapid bactericidal activity due to its ability to sequester surface bound cations, disrupt membrane integrity and lyse bacterial cells. Here we demonstrate that direct contact and the phosphodiester backbone are required for the cation chelating, antimicrobial property of DNA. By treating NETs with excess cations or phosphatase enzyme, the antimicrobial activity of NETs is neutralized, but NET structure, including the localization and function of NET-bound proteins, is maintained. Using intravital microscopy, we visualized NET-like structures in the skin of a mouse during infection with *Pseudomonas aeruginosa*. Relative to other bacteria, *P. aeruginosa* is a weak inducer of NETosis and is more resistant to NETs. During NET exposure, we demonstrate that *P. aeruginosa* responds by inducing the expression of surface modifications to defend against DNA-induced membrane destabilization and NET-mediated killing. Further, we show induction of this bacterial response to NETs is largely due to the bacterial detection of DNA. Therefore, we conclude that the DNA backbone contributes both to the antibacterial nature of NETs and as a signal perceived by microbes to elicit host-resistance strategies.

## Author Summary

Comprising the first line of the innate immune response, neutrophils combat infectious microorganisms through the release of toxic molecules, phagocytosis of invaders and the production of the recently characterized neutrophil extracellular traps (NETs). The antimicrobial activity of NETs has been attributed to proteins bound to the DNA backbone. Our results demonstrate that the DNA lattice of each NET is potently antibacterial and elicits upregulation of protective surface modifications by the opportunistic bacterial pathogen *Pseudomonas aeruginosa*. These modifications, previously shown to protect bacteria from antimicrobial peptides, confer greater bacterial tolerance to DNA and NET-mediated antibacterial activity. Treatments that quench the cation chelating capacity of DNA restore bacterial viability and suppress the expression of surface modifications even in the presence of intact NETs. These observations highlight the dual function of DNA as an antibacterial component of NETs, but also a signal perceived by bacteria to induce broad host resistance strategies. Therefore, the ability of *P. aeruginosa* to sense and defend against the antibacterial activity of neutrophil extracellular traps may contribute to long-term survival in chronic infection sites including the Cystic Fibrosis lung.

## Introduction

Neutrophils are central mediators of the innate immune defense system and perform their role by killing invading microbes through phagocytosis, degranulation, and the release of neutrophil extracellular traps (NETs) [1, 2]. The scaffold of NETs is composed of genomic DNA, which is enmeshed with antimicrobial proteins normally found in the nucleus, granules, or cytoplasm of neutrophils [1, 2]. Although largely characterized *ex vivo* using purified human neutrophils and the chemical inducer phorbol-12-myristate 13-acetate (PMA), the process of NETosis resulting in the generation of NETs has been observed *in vivo* during infection where these structures function to trap bacteria, fungi, protozoa and viruses [1–5]. The mechanism by which NETs kill microbial invaders remains controversial [5–7]. Given the detection of known antimicrobial proteins that decorate the genomic lattice structure [1, 2, 8, 9], current models describing the antimicrobial function of NETs focus on the role of NET-bound proteins. However, most of these proteins are present in low abundance and evidence of their antimicrobial function while bound to the NET structure is limited to a few proteins [1, 8–11]. NET-bound calprotectin is a zinc-chelating protein with antimicrobial activity against *Candida* and *Klebsiella* that can be neutralized with excess zinc [3, 9, 10]. Histones, the most abundant NET-bound proteins [10], possess direct membrane-acting antibacterial activity [12, 13] and were shown to contribute to killing of *Staphylococcus* and *Shigella* [1]. Cathepsin G, a granular serine protease, is required for the clearance of *Neisseria* by NETs [8]. To demonstrate the antibacterial contribution of the latter two NET-bound proteins, antibodies raised to histones or cathepsin G were shown to limit the bactericidal capacity of NETs towards these pathogens [1, 8].

Immunocompromised individuals, including those with Cystic Fibrosis (CF), are particularly susceptible to *P. aeruginosa* infection, which is a major cause of morbidity and mortality in these patients. Chronic *P. aeruginosa* infection of the CF lung leads to an intense inflammatory immune response, resulting in the recruitment of large numbers of neutrophils to the site of infection [14, 15]. CF sputum is highly enriched in neutrophil-derived DNA, including that of NET origin, indicating that neutrophils deploy NETs in an effort to combat infection of the lung [16, 17]. However, persistence of *P. aeruginosa* in the midst of sustained neutrophil presence and NETosis suggests that the pathogen is capable of evading this host immune response [18].

An important virulence strategy adopted by successful microbial pathogens is the tolerance of NETs. In a number of cases described so far, microbial invaders accomplish this goal by either avoiding or disarming neutrophil extracellular traps. Modification of the bacterial capsule and surface-localized lipoteichoic acid reduces the trapping of *Streptococcus pneumonia* in NETs [19]. The secretion of extracellular nucleases by *Staphylococcus aureus*, *Streptococcus pneumonia*, group A *Streptococcus* and *Vibrio cholerae* highlight a shared virulence strategy that functions to degrade the DNA-backbone of NETs, enabling evasion or liberation of the bacteria from entrapment [2, 20–22].

Although microbial or exogenous DNase is proposed to dissolve NET structures to avoid capture, it has not been considered that the DNA backbone itself may be antimicrobial. We have previously demonstrated that extracellular DNA is an efficient chelator of divalent metal cations [23]. This cation chelation has pleiotropic effects on *P. aeruginosa* depending on the concentration of extracellular DNA. At subinhibitory levels, sequestration of cations by DNA leads to the expression of genes controlled by the two component systems PhoPQ and PmrAB that sense Mg^2+^ limitation [23–25]. However, at higher concentrations, extracellular DNA causes dramatic disruptions to the bacterial envelope integrity, which leads to lysis and rapid cell death [23]. It is predicted that the phosphodiester backbone is required for cation sequestration of metal cations on the bacterial surface and the membrane-destabilizing antimicrobial activity of extracellular DNA [23]. Therefore, we hypothesize that the DNA backbone of NETs contributes to their antibacterial activity. Here we show that neutralizing the membrane destabilizing activity of extracellular DNA by quenching the capacity of the phosphodiester backbone to chelate cations protected bacteria from NETs. *P. aeruginosa* is capable of detecting the DNA lattice of NETs, and in response, upregulates genes required to modify the bacterial outer membrane surface to tolerate the toxic effects of DNA-mediated NET killing.

## Results

### *Pseudomonas aeruginosa* induces and tolerates NETosis

**Figure 1:**
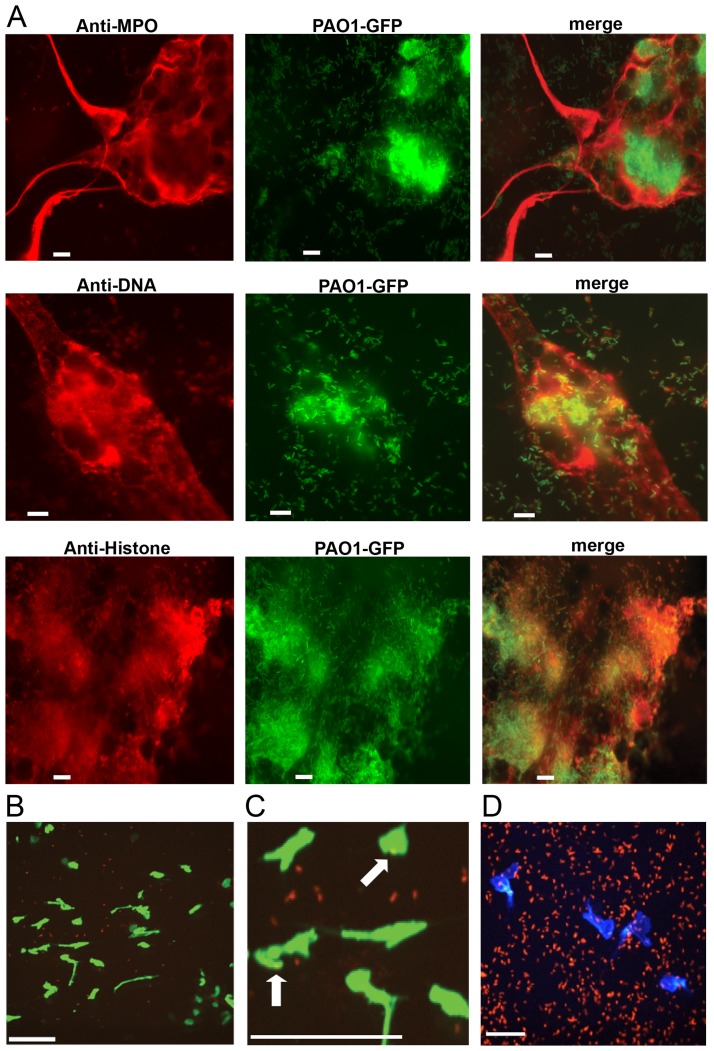
*P. aeruginosa* PAO1 is trapped by human and mouse neutrophil extracellular traps. (**A**) PMA-induced NETs trapped *P. aeruginosa* Tn*7*::*gfp* and contained myeloperoxidase (MPO), DNA and histones when visualized by immunofluorescence with antibodies from autoimmune patient sera (see Methods). Representative images of the NET components (left), Gfp-tagged PAO1 (middle), and the merged (right) are presented. (**B**) NETosis in the skin of mice infected with ChFP-labeled *P. aeruginosa* as visualized by Sytox green stained extracellular DNA structures. Scale bar: 40 μm. (**C**) Arrows indicate ChFP-labeled *P. aeruginosa* trapped by Sytox green-stained NETs *in vivo*. (**D**) ChFP-labeled *P. aeruginosa* is phagocytosed by neutrophils during a skin infection model. Neutrophils (blue) are visualized with anti-mouse GR-1 antibody. Scale bar: 25 μm.

*P. aeruginosa* has been shown to induce the formation of neutrophil extracellular traps in purified human neutrophils [18] and NETs have been observed in CF sputum [16, 17], where *P. aeruginosa* is a predominant pathogen. PMA-induced NETs contained known neutrophil proteins embedded in the DNA lattice, including myeloperoxidase (MPO) and histones [2] (Fig. 1A). We also show that Gfp-tagged *P. aeruginosa* is efficiently trapped and aggregated in NETs (Fig. 1A). However, *in vivo* NETosis during *P. aeruginosa* infection has not been reported. Therefore intravital confocal microscopy was used to determine whether *P. aeruginosa* elicited NETosis in the mouse skin infection model [26]. Infection with *P. aeruginosa* led to the production of large NET-like structures that stained with the DNA-binding dye Sytox green and entrapped ChFP-labeled *P. aeruginosa* (Fig. 1B and C). In addition to the presence of NETs, neutrophils remained chemotactic and phagocytosed bacteria, suggesting that multiple neutrophil clearance mechanisms are employed *in vivo* to combat *P. aeruginosa* (Fig. 1D and S1 Movie).

**Figure 2:**
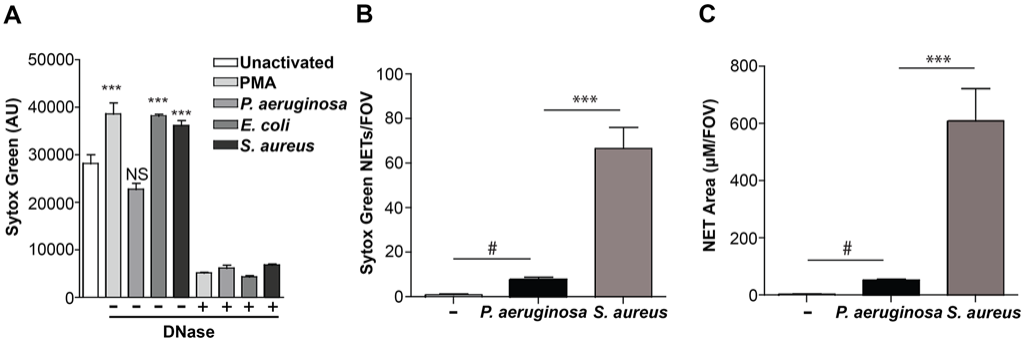
Quantification of NETosis in human neutrophils *ex vivo* and mouse neutrophils during an infection. (**A**) Human neutrophils were stimulated with PMA, *P. aeruginosa*, *E. coli* and *S. aureus* and Sytox green fluorescence was measured as an indicator of DNA release by NETosis after 1 hr stimulation. Exogenous DNase was added as a control to confirm extracellular DNA presence in NETs, indicated by a plus sign (+). Asterisks denote a significant difference in extracellular DNA release between stimulated and unstimulated neutrophils (white bar) (**P<0.01, ***P<0.001). Each value shown is an average from 6 replicates with error bars representing the standard error. (**B**) The total number of NETs in uninfected mice, or infected mice with *P. aeruginosa* PAO1 or *S. aureus* and (**C**) the total NET area quantified. # denotes a significant difference in NET area and number in *P. aeruginosa* PAO1 infected mice compared to the uninfected control (#P<0.05). Asterisks denote a significant difference in NET area and number in mice infected with *P. aeruginosa* compared to *S. aureus* infected mice (***P<0.001).

Given that *P. aeruginosa* induces the production of NETs *in vitro* and *in vivo* (Fig. 1), we sought to compare the relative abilities of *P. aeruginosa*, *S. aureus, E. coli* and the chemical inducer PMA to elicit NETosis. In the presence of purified human neutrophils, PMA, *E. coli* and *S. aureus* induced significantly more NET formation, relative to *P. aeruginosa* within 1 hour of coincubation (Fig. 2A). However, at later time points (3h), *P. aeruginosa* elicited the formation of similar amounts of NET structures. Furthermore, quantification of the number of NETs and NET-area using the skin infection model confirmed that *P. aeruginosa* weakly induces NETosis relative to *S. aureus in vivo* (Fig. 2B and C).

One possible explanation for the reduced NET formation in the presence of *P. aeruginosa* is the production of a microbial secreted DNase that degrades NETs more efficiently than other organisms. Therefore, we measured the DNase activity in overnight culture supernatants, as well as in supernatants isolated from coincubating bacteria with PMA-treated neutrophils. We demonstrate that there was little, if any, DNase activity produced by *P. aeruginosa*, *S. aureus* or *E. coli* under these conditions (S1 Fig.). Since cations are a requirement for DNase activity, we were able to restore DNase activity in *S. aureus* supernatants after the addition of excess cations (S1 Fig.). However, under the cation-free conditions used to quantitate antibacterial NET function, even in the presence of PMA-stimulated neutrophils, DNase activity was not detected and thus *P. aeruginosa* appears to limit NETosis through an uncharacterized mechanism.

**Figure 3:**
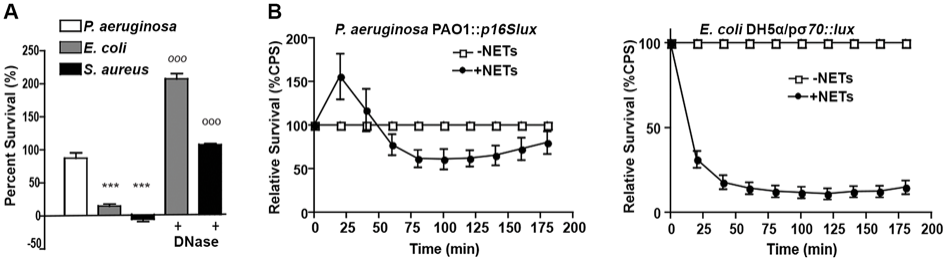
*P. aeruginosa, E. coli and S. aureus* differ in their ability to tolerate the bactericidal effects of NETs. (**A**) Survival analysis of 1 × 10^7^ CFU *P. aeruginosa, S. aureus* and *E. coli* upon exposure to NETs (MOI 10:1). Bacterial viability was determined by direct plate counts (CFU/ml) before and after 4 hour incubation with PMA-activated neutrophils and was normalized to bacterial counts in the absence of neutrophils. DNase I was added exogenously 0.5 hour prior to the end of the experiment to degrade NETs and ensure accurate counts of recoverable colonies. Results are representative of three independent replicates. ***P<0.001 versus *P. aeruginosa* PAO1. °°°P<0.001 versus non-DNase condition by one-way ANOVA with Bonferroni post tests. (**B**) Bacterial viability was determined by measuring luminescence from 1 × 10^7^ CFU *lux*-tagged PAO1::p16S*lux* or *E. coli* DH5α/p*σ70-lux* in the absence or presence of PMA-induced NETs (MOI 10:1). Errors bars represent SEM from six replicates. All experiments were performed at least three times.

In order to characterize the bactericidal capacity of NETs, neutrophils were treated with PMA to stimulate maximal NETosis and cytochalasin D to block phagocytosis, thus restricting bacterial killing to extracellular NET function [1, 8, 21, 22]. Importantly, the addition of cytochalasin D had no effect on NETosis induced in PMA-treated neutrophils (S2 Fig.). We used the conventional method of direct bacterial counts to enumerate the number of bacteria before and after challenge with PMA-induced NETs. Direct counts of NET-exposed bacteria revealed that *P. aeruginosa* was most tolerant to NET killing, whereas *S. aureus* and *E. coli* were significantly more sensitive (Fig. 3A). The addition of deoxyribonuclease (DNase) restored bacterial survival of the NET-sensitive organisms *E. coli* and *S. aureus,* confirming that killing was mediated by extracellular NET function (Fig. 3A). Further, the kinetics of bacterial killing by PMA-generated NETs was determined by measuring the loss of luminescence from chromosomally-tagged luminescent *P. aeruginosa* strain, PAO1::p16S*lux* [23], and plasmid-borne luminescent *E. coli* / pσ*70-lux* [27]. This approach confirmed that *P. aeruginosa* was more tolerant to NET killing than *E. coli*, where luminescence rapidly decreased upon neutrophil challenge (Fig. 3B).

### Direct contact and the phosphate backbone are required for the antimicrobial, cation chelating activity of extracellular DNA

The addition of exogenous DNase I and the production of secreted DNases by bacteria are the most effective means to disable NET killing [1, 2, 20, 21]. It is thought that DNase treatment dissolves NET structures, thereby releasing and diluting the antimicrobial proteins bound to NETs. We have previously shown that extracellular DNA has a potent antibacterial activity, as purified salmon DNA (2% w/v) causes several log orders of bacterial killing within minutes and breaks the integrity of both the inner and outer membranes, leading to lysis [23]. DNA is a very efficient cation chelator and the antimicrobial activity of DNA can be blocked with addition of excess divalent metal cations [23]. It is predicted that the cation chelation is mediated by the phosphodiester backbone.

**Figure 4:**
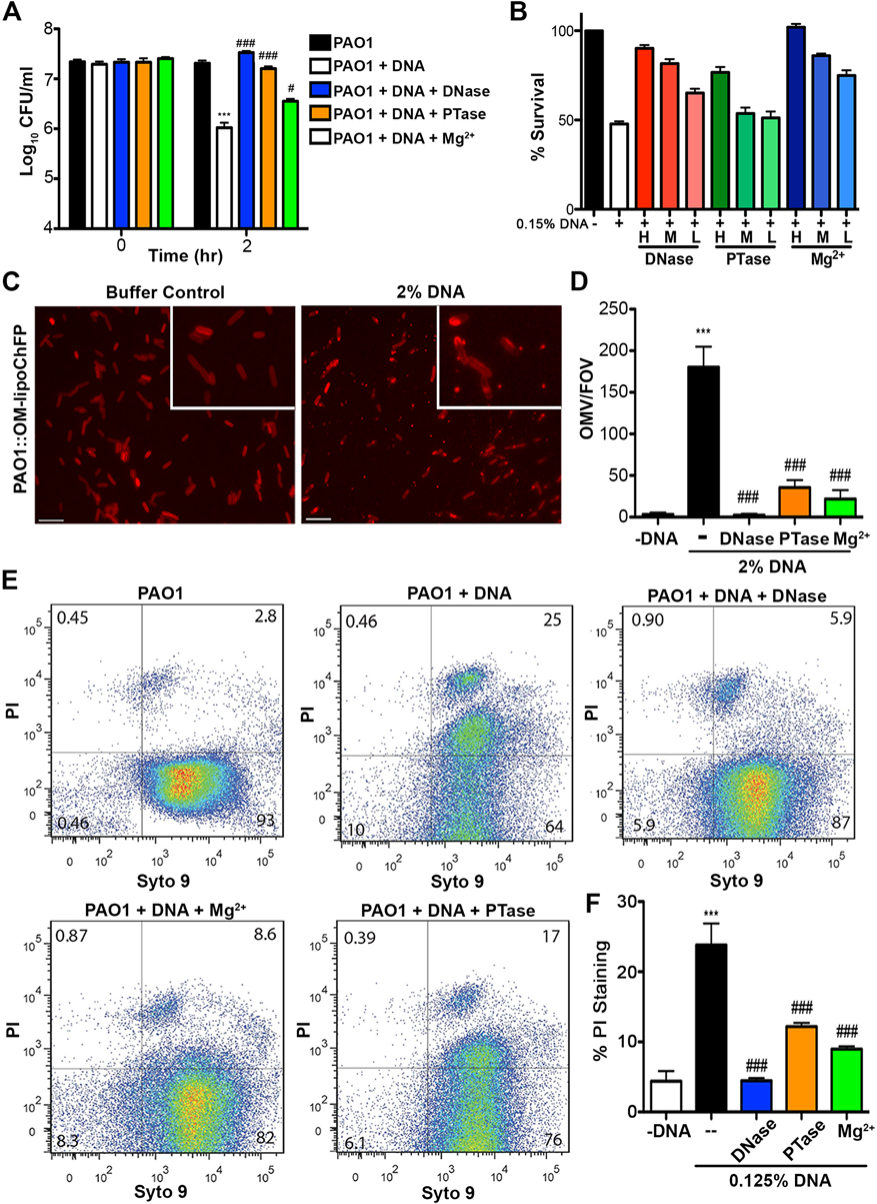
Extracellular DNA exerts bactericidal activity through cation chelation-mediated disruption of the bacterial outer membrane. (**A**) Survival analysis of 1 × 10^7^ CFU *P. aeruginosa* PAO1 coincubated with 0.125% (w/v) DNA or DNA pretreated with DNase I, PTase or 5 mM Mg^2+^. Bacterial counts were performed before (0) and after two hours treatment with DNA (2). Results are representative of three independent experiments. Error bars represent the standard deviation (SD) from eight replicates. (**B**) High, medium and low concentrations of DNase (430kU, 43kU, 4.3kU), PTase (50 U, 10 U, 1 U) or excess Mg^2+^ (5 mM, 500 μM, 5 μM) leads to increased levels of protection from killing with 0.15% DNA (w/v). (**C**) Visualization of the outer membrane integrity of *P. aeruginosa* PAO1::OM-lipoChFP expressing an outer membrane-localized mCherry fluorescent lipoprotein [38] immediately after 2% (w/v) DNA-exposure. Insets represent increased magnification of presented micrographs. Scale bar: 10 μM. (**D**) Quantification of ChFP-rich OMV generation in the field of view from 6 representative images generated from bacteria-DNA coincubation as described in (**C**) or DNA pretreated with DNase, PTase and Mg^2+^. Error bars represent SD from 6 fields of view. (**E**) Flow cytometry of DNA-exposed *P. aeruginosa* PAO1 using SYTO9-PI dual staining as a measure of membrane-compromised bacteria [28, 32]. 2.5 × 10^7^ CFU *P. aeruginosa* PAO1 were exposed to 0.0125% DNA alone or pretreated as in (**A**) then immediately analyzed by the collection of positive events (N = 50 000) by BD LSRII. Numbers in corners represent the % of 50 000 events that fall into each quadrant gate. (**F**) Quantification of membrane-compromised, PI-stained *P. aeruginosa* PAO1 as measured by flow cytometry. Mean percent PI stained was derived from the average of three replicates (each with N = 50 000 for each plot) in each exposure condition as in (**E**). *** denotes a significant difference between the control and 0.125% DNA sample. ### and # denote a statistically significant difference, P<0.01 and P<0.05, respectively, between DNA alone sample and pretreated samples. Two-tailed student t-tests were performed to test for significant differences.

To confirm the mechanism by which extracellular DNA kills bacteria, we monitored the loss of *P. aeruginosa* viability in the presence of dilute extracellular DNA (0.125% w/v; Fig. 4A). Bacterial survival was restored if DNA was pretreated with DNase or excess 5mM Mg^2+^, which degrades DNA or saturates its cation chelating ability, respectively, thus neutralizing the antibacterial activity (Fig. 4A). Pretreatment of extracellular DNA with calf intestinal alkaline phosphatase (PTase), which cleaves 5’-phosphates, also blocked the observed antibacterial activity (Fig. 4A). The addition of decreasing amounts of DNase, PTase or Mg^2+^, resulted in marked, dose-dependent, decreases in bacterial survival when challenged with 0.15% DNA (Fig. 4B). The DNA-mediated damage to the outer membrane leads to the formation of ChFP-enriched outer membrane vesicle-like structures (OMVs; Fig. 4C) [23]. However, incubation of *P. aeruginosa* with extracellular DNA pretreated with DNase, PTase and Mg^2+^ greatly diminished the number of ChFP-labeled OMVs relative to control conditions (Fig. 4D), confirming that the bactericidal mechanism of extracellular DNA is through disruption of the bacterial membrane.

Extracellular DNA-mediated damage to membrane integrity was confirmed by using flow cytometry (Fig. 4E). Treatment of *P. aeruginosa* with DNA resulted in a new population of cells that were dual positive for SYTO9 and PI, indicative of membrane damage and increased PI uptake, and possibly dead cells. The increased staining of DNA-exposed bacteria by membrane-impermeable propidium iodide (PI) was not observed in DNase and Mg^2+^ pretreatments and was reduced in PTase pretreated DNA samples (Fig. 4E and F). To ensure cation chelation was responsible for the observed bacterial membrane destabilization, we assessed whether the known cation chelator EDTA could cause membrane disruption. Like DNA, EDTA caused major outer membrane disruptions and the release of OMVs, as well as a dramatic increase in PI-staining of EDTA-treated cells monitored by flow cytometry (S3 Fig.) [28]. To determine whether the antibacterial capacity of DNA requires direct bacterial contact or can be mediated through passive cation sequestration, we exposed *P. aeruginosa* to high concentrations of DNA spatially separated by an ion-permeable barrier. Blocking direct interaction between *P. aeruginosa* and DNA resulted in bacterial survival after a prolonged exposure, compared to the rapid antibacterial activity of DNA in direct contact with the bacteria (S4 Fig.). Together, these results demonstrate that the antibacterial activity of extracellular DNA requires direct contact, and the phosphate backbone for cation chelation, leading to membrane disruption and bacterial cell death.

### Bactericidal activity of NETs is blocked by neutralizing the cation chelation capacity of DNA

**Figure 5:**
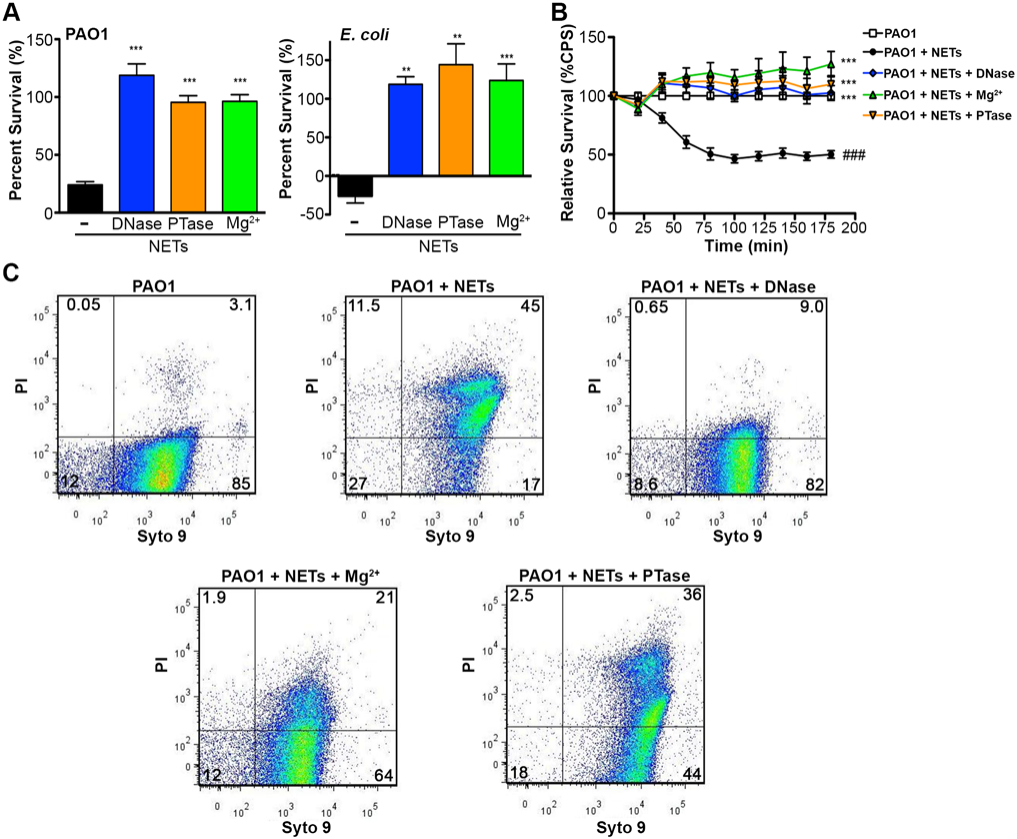
Neutralizing the cation chelating activity of the DNA backbone of NETs protects bacteria. (**A**) Percent survival of *P. aeruginosa* PAO1 and *E. coli* DH5α as determined by direct plate counts (CFU/ml) before and after 4 hour incubation with PMA-activated neutrophils or combined treatment of NETS with DNase, PTase or Mg^2+^. Error bars are SEM from 6 replicates. ** or *** denotes a statistically significant difference (P<0.05 or P<0.01, respectively) between NET-alone versus NET and enzymatic or excess cation treatments, as determined by one-way ANOVA with Bonferroni post tests. (**B**) Luminescence-based viability as a real-time measure of *P. aeruginosa* PAO1::p16S*lux* survival in the presence of NETs alone, or combined treatment of NETS with DNase I, PTase or Mg^2+^. ### denotes a statistically significant difference of P<0.001 between NET-challenged PAO1 versus PAO1 alone (white). ***P<0.001 versus NET killed samples (black). (**C**) Flow cytometry of *P. aeruginosa* PAO1 (2 × 10^7^ CFU) coincubated for four hours with PMA-stimulated neutrophils alone (1 × 10^6^; MOI: 10) or with the addition of DNase I, PTase and 5 mM Mg^2+^. N = 50 000 for each plot. Numbers in each corner represent the % of 50 000 events that fall into each quadrant gate.

The bactericidal activity of neutrophil extracellular traps is attributed to direct contact and exposure of bacteria to the antimicrobial proteins embedded in the DNA scaffold of NETs [1, 2]. Given the antimicrobial activity of DNA, we propose that the DNA backbone of the NET itself is antibacterial. Therefore, if DNA contributes to bacterial killing, treatments that quench the cation chelation potential of the DNA backbone will block bactericidal activity of NETs. To address this possibility, PMA-activated neutrophils were treated with the addition of DNase, PTase or excess Mg^2+^ and bacterial viability was monitored. The DNA-targeted treatments completely protected *P. aeruginosa* and *E. coli* from killing by neutrophil extracellular traps (Fig. 5A). To confirm these results, we monitored the luminescence of *P. aeruginosa* PAO1::p16S*lux* co-incubated with PMA-activated neutrophils and observed that the antibacterial effects of NETs were neutralized by treatment with exogenous DNase, Mg^2+^ cations and PTase (Fig. 5B).

**Figure 6:**
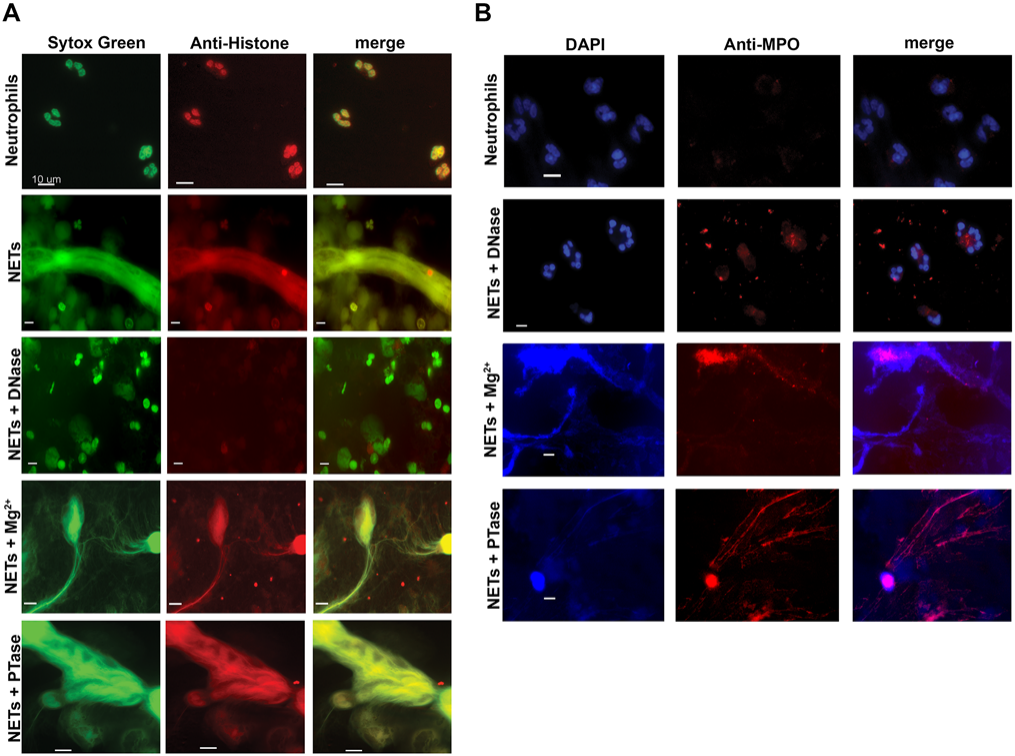
Structurally intact NETs still possess histones and MPO after treatment with excess Mg^2+^ and PTase. Neutrophil extracellular traps were visualized with (**A**) Sytox Green staining of DNA and anti-histone primary antibodies. (**B**) Neutrophil extracellular traps were visualized with DAPI staining of DNA and anti-MPO primary antibodies. NETs were not observed in unactivated neutrophils, but were present in PMA-induced NETs that were treated with either excess 5 mM Mg^2+^ or exogenous PTase. Alexa Fluor 647-conjugated secondary antibodies were used to visualize histone H1 and MPO. Representative immunofluorescence images were merged to show overlap of histone H1 and MPO with structurally intact NETs. Scale bars, 10 µm.

To determine whether the restored bacterial viability in the presence of NETs was due to preventing damage to the bacterial envelope, we performed flow cytometry to assess membrane integrity. Increased PI staining of NET-exposed bacteria is an indicator of membrane damage, which was completely blocked by addition of DNase (Fig. 5C). The addition of excess Mg^2+^ or treatment with exogenous PTase also limited membrane damage (Fig. 5C). Importantly, both Mg^2+^ and PTase treatments neutralized the antimicrobial activity of NETs (Fig. 5A and B) did not disrupt overall NET architecture, as MPO and histones were still present within the treated NET structures (Fig. 6). To assess the function of a NET-bound protein, we measured elastase activity in PMA-treated neutrophils and showed no difference in elastase activity when NETs were treated with exogenous PTase or Mg^2+^ (S5 Fig.). NET structures remain intact and contain functional proteins (elastase) after treatment with PTase or excess Mg^2+^, but are no longer antibacterial for *E. coli* and *P. aeruginosa* (Fig. 5). Together these results suggest an antibacterial mechanism wherein the DNA backbone of NETs target and destabilize the bacterial membrane and promotes cell death.

### Extracellular DNA elicits induction of surface modifications that protect *P. aeruginosa* from NETs

**Figure 7:**
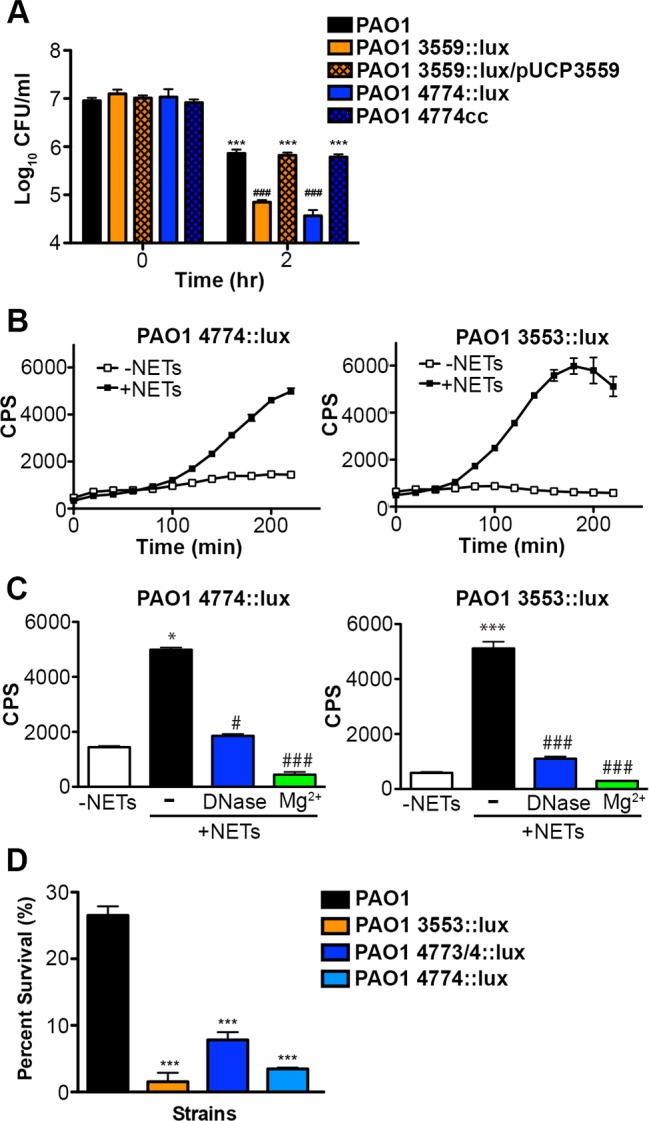
*Pseudomonas aeruginosa* responds to DNA in neutrophil extracellular traps and induces protective bacterial surface modifications. (**A**) Survival analysis of 1 × 10^7^ CFU wild-type *P. aeruginosa* PAO1 or mutants with defects in the aminoarabinose-LPS modification (*PA3553::lux*) or in spermidine synthesis (*PA4774::lux*) after coincubation with 0.125% DNA. *** denotes a statistically significant difference between time 0 and 2 hours post coincubation with 0.125% DNA. ### denotes a statistically significant difference of P<0.001 between wild-type and mutant *P. aeruginosa* when exposed to 0.125% DNA. Two-tailed student t-tests were performed to test for significant differences. Error bars represent SD from eight replicates. (**B**) 2 × 10^7^ CFU *P. aeruginosa* PAO1 spermidine synthesis (*PA4774::lux*) and aminoarabinose modification (*PA3553::lux*) transcriptional reporter strains were incubated with PMA-activated neutrophils (MOI 10:1) and gene expression (luminescence as quantified by CPS) was measured every 20 minutes in the absence (empty squares) and presence (solid circles) of NETs. Error bars represent SEM from six replicates. (**C**) The effect of DNase or 2 mM Mg^2+^ treatment on NET-mediated gene induction of 2 × 10^7^ CFU *PA4774::lux* or *PA3553::lux* after four hours of coincubation (MOI 10:1). *P<0.05, ***P<0.001 versus bacteria alone (white bar), #P<0.05, ###P<0.001 versus NET exposure (black bar) as determined by one-way ANOVA with Bonferroni post tests. (**D**) Bacterial survival analysis of 2 × 10^7^ CFU NET-exposed *P. aeruginosa* PAO1 wild-type, aminoarabinose modification mutant *PA3553::lux*, or the spermidine synthesis mutants *PA47743/4::lux*, *PA4774::lux* (MOI 10:1) Error bars represent the SEM from 6 replicates. *** denotes a statistically significant difference of P<0.001 versus wild-type survival as determined by one-way ANOVA with Bonferroni post tests. All assays were conducted at least three times and representative data is presented.

Subinhibitory concentrations of extracellular DNA sequester Mg^2+^ and trigger the expression of multiple surface modifications that are known to protect the bacterial outer membrane from antimicrobial peptide (AP) damage and killing [23–25]. The *arn* operon (*PA3552-PA3559*) is required for the covalent addition of aminoarabinose to the phosphates of lipid A and the spermidine synthesis genes (*PA4773-PA4774*; *speDE* homologs) are required for production of the polycation spermidine on the outer surface [23, 24]. Both modifications substitute for divalent metal cations, mask the negative charges of the outer surface and thus contribute to AP resistance [23, 24, 29–31]. Given that these modifications stabilize the bacterial envelope, we sought to determine whether these surface modification pathways provided a more general mechanism to resist bacterial membrane damage. We noted that *P. aeruginosa* strains with mutations in the *arn* or spermidine biosynthetic pathways were significantly less capable of surviving exposure to DNA (Fig. 7A).

Given the role of these pathways for tolerance of exogenous DNA, we then investigated whether the DNA component of NETs induced expression of the *arn* or spermidine synthesis genes in *P. aeruginosa*. Expression of both pathways was strongly induced 2–6 fold following co-incubation with NETs produced by PMA-activated neutrophils (Fig. 7B). To confirm that DNA was the component of NETs that led to induction of the bacterial gene expression response, the addition of excess Mg^2+^ cations and enzymatic treatment with DNase (Fig. 7C) and PTase (S6 Fig.) all blocked the induction of the *arn* and spermidine operons response to NETs. While these treatments specifically neutralize DNA, we also considered the possibility that NET-bound antimicrobial proteins including histones or LL-37 may elicit these protective responses. We have previously shown that sub-MIC concentrations of antimicrobial peptides induce both outer surface modifications [29]. Therefore, to assess the relative capacity of each NET component to act as a bacterial signal, we compared the ability of purified histones, the well-characterized APs polymyxin B and colistin, and DNA to induce the expression of the spermidine synthesis pathway. Although all NET components induced expression of the *PA4773-PA4774* spermidine synthesis pathway (S7 Fig.), DNA was the most potent inducer of this bacterial response (S7 Fig.).

**Figure 8:**
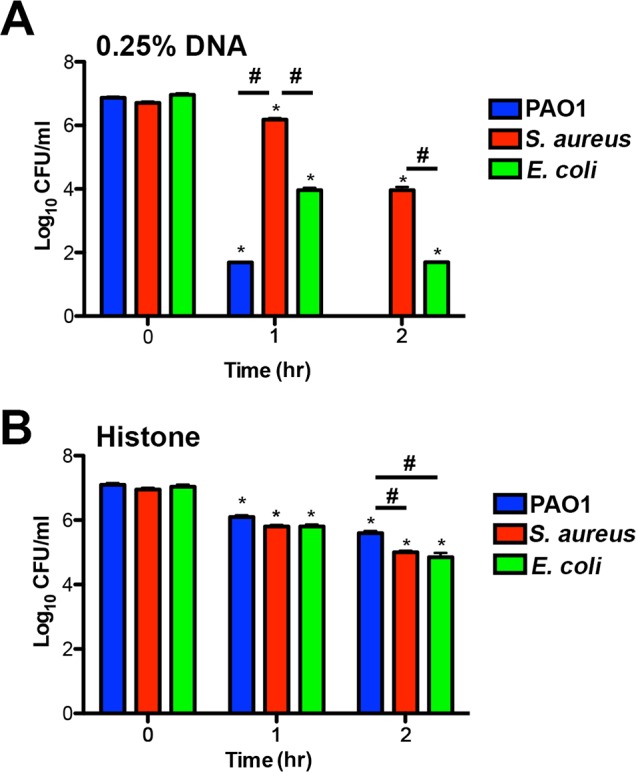
Bacterial species differ in their susceptibility to killing by DNA and histones. (**A**) *P. aeruginosa* PAO1, *S. aureus* and *E. coli* killing assay in the presence of 0.25% DNA (w/v). PAO1 was significantly more sensitive at both eDNA concentrations relative to the highly tolerant *S. aureus*. (**B**) *P. aeruginosa* PAO1, *S. aureus* and *E. coli* killing assay in presence of 1.25 µg/mL histones. Bacterial survival was quantified after 1 and 2 hours by colony count, and statistical significance assessed by 2-tailed student t-tests. * denotes statistical differences (P<0.05) between the indicated time point and the initial bacterial count, while # indicates significant differences (P<0.05) between bacterial species. The values shown are the mean plus standard deviation from 8 samples. Experiments were repeated three times and the data shown is from one representative experiment.

The upregulation of protective outer membrane modifications (aminoarabinose-modified LPS and surface spermidine production) by NETs, and by the individual NET components of DNA and histones, suggests that these modifications are required to defend the membrane against assault from multiple innate immune components that target the bacterial membrane. Both modifications result in stable substitutions for divalent metal cations in the outer membrane, and protect *P. aeruginosa* from antimicrobial peptides [23, 24, 29–31] and DNA killing (Fig. 7A). *P. aeruginosa* mutants in the *arn* and spermidine synthesis genes also exhibited increased susceptibility to the disruptive effects of NETs (Fig. 7D). We next compared the susceptibilities of *P. aeruginosa*, *E. coli* and *S. aureus* to histone and DNA killing. Surprisingly, *P. aeruginosa* was the most susceptible to DNA killing, while *S. aureus* was the most tolerant, the opposite pattern of NET susceptibility (Figs. 3A and 8A). The bactericidal capacity of purified histones was modest, where after 2 hours exposure *P. aeruginosa* was the more histone tolerant (Fig. 8B). Taken together, these results highlight the ability of *P. aeruginosa* to quickly respond and defend against the DNA and histone mediated-antibacterial effects of NETs by stabilizing the outer membrane.

## Discussion

Given the observations that exogenous or secreted microbial DNases protect bacteria against NET killing and that extracellular DNA has rapid, membrane-damaging antibacterial activity, we sought to test the hypothesis that the DNA backbone of NETs contributes to their bactericidal function. Extracellular DNA possessed contact-mediated antibacterial activity that could be neutralized by enzymatic and cationic treatments that degrade or quench the capacity of DNA to chelate cations (Fig. 4 and S3 Fig.). NETs exposed to the same treatments that target the DNA scaffold were unable to cause bacterial membrane damage or to cause bacterial killing of *P. aeruginosa* and *E. coli* (Fig. 5). Therefore, we propose a novel bactericidal mechanism of NETs whereby the removal of surface-stabilizing cations by the DNA phosphodiester backbone results in bacterial lysis (Fig. 4B and C). To address the controversy surrounding the bacterial killing ability of NETs [5–7], we used multiple viability assays to measure NET killing and membrane damage, which included direct bacterial counts, luminescence viability assays and flow cytometry of PI-stained cells [32]. Taken together, these data support the general notion that NETs are directly antimicrobial.

Deciphering the specific antimicrobial mechanisms of NETs has been limited to a few candidate proteins [1, 8–11]. Most studies have focused on the NET-bound proteins as the antimicrobial components, given their important role during phagocytosis and degranulation. Although granular, cytoplasmic and nuclear proteins derived from neutrophils can be detected in NETs by immunofluorescence, the abundance of most NET-bound proteins is low (<1–6%), relative to histones, which comprise 65% of the total protein content [10]. Although classically characterized as chromatin structural proteins, NET-bound histones possess antimicrobial activity that can be neutralized through the addition of anti-histone antibodies [1, 12, 13]. However, other neutrophil proteins exhibit altered or reduced enzymatic activity when enmeshed in the NET backbone raising questions as to their antimicrobial capacity. For example, *S. aureus* tolerates myeloperoxidase in NETs unless supplemented with the addition of exogenous H_2_O_2_ [11]. Neutrophil elastase activity increases in DNase treated sputum from Cystic Fibrosis patients, suggesting that DNA may inhibit elastase activity, thus limiting the role of elastase as a potential NET-bound factor [17].

We observed that both the antibacterial activity of NETs and the ability to induce protective bacterial responses were blocked by treatments that target extracellular DNA, suggesting that the NET scaffold is not simply a passive structure (Figs. 5 and 7) [23–25, 31]. The most potent inducing triggers of the *P. aeruginosa* surface modifications are purified eDNA, followed by APs and purified histones (S7 Fig.). Since being widely induced by these components, it is not surprising that the aminoarabinose-modified LPS and spermidine synthesis pathways protect the outer membrane from DNA, NETs (Fig. 7) and antimicrobial proteins [23, 24, 29–31]. The low potency of the tested histones may be explained by the fact that our assays were performed with a mixture of full-length histones, which had modest antibacterial activity (Fig. 8). Recent evidence highlights that histones are proteolytically processed by proteases such as elastase during the process of nucleus decondensation, prior to NET release [33] It is therefore likely that potent bactericidal histone-derived peptides are present in NETs as an important antibacterial component of NETs.

*P. aeruginosa* appears to mount a multifunctional, outer membrane defense strategy to combat multiple antimicrobial components enmeshed in NETs. Consistent with this model, we noticed unexpected susceptibility patterns that also suggest that NET killing may be the result of the combinatorial effect of DNA and NET-bound proteins. The observation that *S. aureus* is susceptible to NET killing but tolerant to DNA (Figs. 3 and 8), suggests that NET killing of *S. aureus* is likely dependent on other anti-staphylococcal proteins enmeshed in the DNA lattice. The DNA susceptibility phenotype of *P. aeruginosa* may explain the potency of DNA as the strongest inducer of the protective outer surface modifications that contribute to the observed NET tolerance. It is intriguing to speculate that the NET-bound, antimicrobial proteins act in concert with the antibacterial activity of DNA to provide broad-spectrum protection against a wide range of microbial pathogens.

Modification of the bacterial cell surface and the production of secreted DNases are virulence strategies utilized by microbial pathogens to evade NET killing [19–22]. Here we report that the spermidine and the *arn* surface modification pathways are required to tolerate the antibacterial action of both DNA and NETs (Fig. 7). The covalent addition of aminoarabinose to the lipid A component of LPS masks the negative charges of core LPS phosphates, and the polycationic nature of spermidine (+3 charge) substitutes for surface divalent metal cations, and may also bind and neutralize DNA. In addition, spermidine possesses an antioxidant activity that protects bacterial membrane lipids from oxidative damage [24] and therefore may protect *P. aeruginosa* from NET-induced oxidative damage [11]. Combined, these results suggest that the spermidine and *arn* surface modifications possess multiple protective roles that may contribute to resisting a broad range of antimicrobial components present within NETs.

We propose that bacterial surface-bound, divalent metal cations are displaced by direct contact with extracellular DNA, and that DNA-induced surface modifications prevent outer membrane disruption and bacterial killing by NETs. Therefore, the antibacterial mechanism of cation chelation exerted by DNA is distinct from that of other previously characterized antimicrobial cation chelating proteins such as calprotectin. We have previously demonstrated that DNA chelates diverse metal cations (Mg^2+^, Ca^2+^, Zn^2+^, Mn^2+^) [23] while calprotectin chelates zinc and manganese [34]. Additionally, the antimicrobial function of calprotectin is contact-independent whereas the bactericidal function of DNA requires contact (S4 Fig.). Further, sequestration of zinc and manganese by calprotectin does not target the microbial membrane but rather sequesters cation cofactors required by bacterial enzymes such as superoxide dismutase, which protects bacteria from superoxide [34].

In summary, we have identified that the DNA backbone is a *bona fide* antibacterial component of neutrophil extracellular traps. The DNA scaffold structure also acts as a warning signal perceived by *P. aeruginosa*. Overall, these results support a model where the membrane-destabilizing activity of the DNA scaffold contributes to the bactericidal capacity of NETs, while the cation chelating activity acts as a signal perceived by *P. aeruginosa* that leads to upregulation of protective surface modifications. These results highlight a dynamic bacterial-host interaction between an opportunistic pathogen that causes chronic infections in the lungs of individuals with Cystic Fibrosis, an infection site known to be rich in neutrophil DNA and neutrophil extracellular traps [16, 17]. This ability to sense and defend against NETosis may help explain the long-term persistence of *P. aeruginosa* in CF lung infections.

## Materials and Methods

### Bacterial strains and growth conditions

All strains and plasmids used in this study are shown in S1 Table. Bacterial cultures were routinely grown at 37°C in LB or BM2 defined minimal media with 0.5 mM MgSO_4_, unless otherwise stated. *S. aureus* was grown overnight in BHI media. When necessary, the following antibiotics were used: 50 µg/mL tetracycline for *P. aeruginosa* mini-Tn *5-lux* mutants, and 50 µg/mL kanamycin for *E. coli* DH5α/ pσ70-*lux*. Mid-log cultures were used for co-incubation experiments with neutrophils or extracellular DNA.

### Human neutrophil isolation

Neutrophils were isolated from healthy donors as previously described [35]. Whole blood was collected and mixed 5:1 in acid citrate dextrose, followed by removal of red blood cells using dextran sedimentation and hypotonic lysis with KCl. After all red blood cells were lysed, the cell pellet was subjected to Ficol-Histopaque density centrifugation. The subsequent pellet was resuspended in 2 mL of HBSS (Hank’s balanced salt solution, no cations; Invitrogen 14175–095). The viable cell concentration was determined using a haemocytometer and Trypan blue staining.

### NET imaging

Glass cover slips were HAS-coated and placed in 6-well tissue culture plates. Neutrophils were added at 2.0×10^6^ cells/mL per well, adhered (30 min, 5% CO_2_, 37°C) and treated with cytochalasin D (10 µg/well) and PMA (25 nM) to activate NETosis [35]. Mid-log bacterial cultures were diluted in HBSS (no cations) (5.0x10^7^ CFU/mL) for an MOI of 25:1, centrifuged to the neutrophils, and coincubated for 1–4 hours (5% CO_2_, 37°C). Cells on cover slips were fixed with 4% paraformaldehyde, washed with 250 µL of 10% FBS (Invitrogen) in PBS and stained with either DNA dyes and/or various primary and secondary antibodies (described below).

### Immunofluorescence microscopy of NETs

For NET visualization with antibodies, the primary anti-human MPO antibody (DakoCytomation- A0398) was diluted into 10% FBS in PBS (1/500). 30 µL was added to adhered neutrophils, incubated (30 min, 37°C) and washed twice with sterile PBS. 40 µL of the secondary anti-rabbit Cy 5 antibody (Jackson ImmunoResearch 60354) (1/500 dilution) was added. After 15 min incubation in the dark, cover slips were washed twice with PBS, and prepared with mounting media. Anti-DNA and anti-histone antibodies where obtained from Dr. Marvin Fritzler. Either the anti-DNA (1:10) or anti-histone (1:500) antibodies [36, 37] were added as described above. The anti-human secondary antibodies with Alexa Flour 647 (Invitrogen A21445, 1/500) were added to cover slips and mounted as described above. Images of human NETs were acquired using the Leica DMI 4000B inverted microscope equipped with ORCA R2 digital camera and Metamorph software for image acquisition using the 63X or 100X objectives. The following excitation and emission filters were used for blue fluorescence (Ex 390/40; Em 455/50), red fluorescence (Ex 555/25; Em 605/52), far red fluorescence (Ex 645/30; Em 705/72) and green fluorescence (Ex 490/20; Em 525/36). Images were formatted and analyzed using the Imaris 7.0.0 imaging software. All images shown are representative of at least three experiments.

### Mouse skin infection model

Mice were anaesthetized (10 mg/kg xylazine hydrochloride and 200 mg/kg ketamine hydrochloride) and body temperature was maintained using a rectal probe and heating pad. The mice were pretreated with intradermal MIP-2 (0.2µg/injection) diluted in sterile normal saline 30 minutes prior to imaging. The right jugular vein was cannulated to administer additional anesthetic and fluorescent dyes. The microcirculation of the dorsal skin was prepared for microscopy as previously described [26]. Briefly, after shaving the mouse’s back, a midline dorsal incision was made extending from the tail region up to the level of the occiput. The skin was separated from the underlying tissue, remaining attached laterally to ensure the blood supply remained intact. The area of skin was then extended over a viewing pedestal and secured along the edges using 5.0 sutures. The loose connective tissue lying on top of the dermal microvasculature was carefully removed by dissection under an operating microscope. The exposed dermal microvasculature was immersed in isotonic saline and covered with a coverslip held in place with vacuum grease. Alexa Fluor 649 conjugated anti-mouse GR-1 antibody (10µl per mouse i.v.; eBioscience) was used visualization of neutrophils. To visualize NETs *in vivo* the membrane impermeable dyes SYTOX-green or SYTOX-orange were administered (diluted 1:1000 with sterile saline, 100µl per mouse i.v.). MIP-2 injection (0.2µg/injection) was initiated 30 min prior to *Pseudomonas aeruginosa* or *S. aureus* administration. Following baseline visualization, all bacteria were directly administered into the field of view using a tuberculin needle (1×10^8^ CFU/100µl of sterile saline, i.d.).

### Intravital microscopy

Spinning disk confocal intravital microscopy was performed using an Olympus BX51WI (Olympus, Center Valley, PA) upright microscope equipped with a 20×/0.95 XLUM Plan Fl water immersion objective. The microscope was equipped with a confocal light path (WaveFx, Quorum, Guelph, ON) based on a modified Yokogawa CSU-10 head (Yokogawa Electric Corporation, Tokyo, Japan). Laser excitation at 488, 561 and 649nm (Cobalt, Stockholm, Sweden), was used in rapid succession and fluorescence in green, red and blue channels was visualized with the appropriate long pass filters (Semrock, Rochester, NY). Exposure time for all wavelengths was between 500 and 600ms. Sensitivity settings were maintained at the same level for all experiments. A 512×512 pixels back-thinned EMCCD camera (C9100–13, Hamamatsu, Bridgewater, NJ) was used for fluorescence detection. Volocity Acquisition software (Improvision Inc., Lexington, MA) was used to drive the confocal microscope. Images captured using the spinning disk were processed and analyzed in Volocity 6.0.1. NET area and NET number were quantified using the Volocity software.

### Quantification of NET area and number

NET area was determined using Volocity imaging software. Briefly, in each field of view (FOV) the threshold of the corresponding fluorescent channel in which NET structures were stained was set to eliminate the background staining of the skin. The area and number of NET positive structures in the FOV was calculated and counted via the Volocity 6.0.1 software. Structures that showed no characteristic NET-like shape and resembled the staining of a nucleus of an obvious dead cell in the FOV were excluded from the quantification manually. NET image analysis was performed in at least two infected or uninfected animals and from 5 fields of view.

### NET quantification from purified human neutrophils

NETs were quantitated by measuring the amount of extracellular DNA that stains with the cell impermeant dye Styox Green [35]. Cell culture media (CCM) consisted of 48.5 mL of RPMI 1640 (Invitrogen), 0.5 mL of 1.0 M HEPES and 1.0 mL of human serum albumin (HAS; Innovative Research). Neutrophils were diluted into CCM (2.0×10^5^ cell/well) and added to an HSA-coated, 96-well black, clear-bottom plate (Thermo Scientific). As a positive control, PMA (25 nM/well) was added to activate the neutrophils [35]. For bacterial activation of NETs, mid-log bacterial cultures were diluted in CCM (2.0×10^6^ CFU/well) for a multiplicity of infection (MOI) of 10:1 (bacteria to neutrophils). DNase (430 kU/well; VWR 31149) was added to degrade extracellular DNA in NETs. Bacteria were gently centrifuged onto the adhered neutrophils (800xg, 10 min). Sytox green (2.5 µM; Invitrogen) was added to each well and green fluorescence (Ex 490/8; Em 535/25) was measured with Perkin Elmer 1420 Multilabel Counter Victor^3^ between 1 and 4 hours. All values shown are the mean from at least three individual replicates and each experiment was performed at least 3 times. We noticed variation in the background level of NET staining between individual donors, but there was a reproducible and robust 2.5 to 10-fold increase in NETosis after PMA treatment of neutrophils from various donors (S2 Fig.).

Quantification of Bacterial Viability and Gene Expression Using Plate Counts and Luminescence NET killing was examined using direct plate counting methods where a reduction in cell number indicated bacterial killing. All NET killing experiments were performed in HBSS solution lacking divalent cations. Isolated human neutrophils were mixed with mid-log bacteria in HBSS (no cations) in black, clear-bottom 96-well plates with of 2.0 × 10^7^ CFU bacteria and 2.0 × 10^6^ neutrophils (MOI 10:1). After a 1–4 hr incubation, 50 µL of DNase I solution (430 kU/mL) was added to every well, mixed, and incubated for 30 min at 37°C, in order to release bacteria trapped in NETs for accurate plate counts. 15 µL of suspension was serially diluted (1/10) in 0.9% NaCl solution in a sterile 96-well plate and 5 µL from each well was stamped onto LB agar plates to obtain bacterial plate count data for time zero (T_0_) and after 4h (T_4_). CFU/ml values from T_4_ and T_0_ time points were used to calculate the percentage survival by subtracting the T_4_—T_0_ plate counts and dividing the ‘bacteria and neutrophil’ conditions by the ‘bacteria alone’ conditions and multiplying by 100. For *lux* viability and gene expression assays [23], bacteria were centrifuged onto the adhered neutrophils and placed in the Victor^3^ plate reader for luminescence (CPS) measurements every 20 minutes for 3–4 hours. All values shown are the mean from at least six individual replicates and each experiment was performed at least 3 times.

### Flow cytometry analysis of SYTO9/propidium iodide stained bacteria

SYTO9 stains the DNA in all cells and propidium iodide (PI) stains the DNA in dead cells and cells with damaged membranes [28, 32]. The sample of bacteria and neutrophils (~200 µL) was placed in 5 mL polystyrene round-bottom sample tubes and stained with SYTO9 and propidium iodide at final concentrations of 0.02 mM and 0.2 mM, respectively. The tubes were centrifuged at 300x gravity and incubated (RT, 15 min). Bacterial cells were analyzed using the BD LSRII flow cytometer (BD Bioscience, San Jose, USA) equipped with a blue laser (488nm) and a green laser (532nm). Unstained, mid-log bacterial cells were used to gate the forward scatter (FSC) and size scatter (SSC) parameters. For green and red fluorescence profiles, SYTO9 was excited by blue (488nm) laser with emission filters 525/50BP and 505LP and PI was excited using the green (532 nm) laser with emission filters 610/20BP and 600LP. All detectors were set to the logarithmic amplification with the following voltages, 500, 240, 596, and 489 and threshold was set at 200 for both FSC and SSC. For each sample, 50 000 events were acquired using the BD FACSDiva software 6.1.3. The Hierarchical gating strategy was used to determine double positive population of bacterial cells (stained with both SYTO9 and PI) where gate P1 is the total population of FSC and SSC gated events, as determined from bacteria alone control and then applied to all other samples. P2 is the population of events stained by SYTO9 and P3 is the population stained with both SYTO9 and PI. Neutrophils and mid-log bacteria controls do not contribute any autofluorescence or PI-stained events when stained with either or both of the SYTO9/PI dyes. Values displayed in each density plot represent the percentage of 50 000 cells (N value) in each quadrant gate and each experiment was performed with at least 5 times.

### DNase, PTase or Mg^2+^ treatment of NETs

During the coincubation experiments of bacteria and PMA-activated neutrophils, exogenous Mg^2+^ was added at a final concentration of 5 mM MgSO_4_. For the enzyme treatments, deoxyribonuclease (DNase I, VWR) was added at a final concentration of 430 kU/well and calf intestinal alkaline phosphatase (PTase, Invitrogen) at a final concentration of 16.6 U/well. Maximum enzyme amounts were added to bacterial-neutrophil mixtures that had no effect on bacterial viability and without the addition of enzyme buffers. The killing experiments were incubated for up to 4 hours in the 5% CO_2_ incubator at 37°C. All % survival values shown are the mean from at least three individual replicates and each experiment was performed at least 3 times.

### Extracellular DNA and histone killing assays

*P. aeruginosa* was grown to mid-log in LB medium (OD_600_ = 0.2–0.4), washed and resuspended in 10 mM Tris buffer (pH 7.4; 1.0 × 10^7^ CFU/well). Cells were incubated with fish sperm DNA (0.125%, w/v; USB) or with DNA that had been pretreated with exogenous DNase I (150 kU/well), PTase (50 U/well) or 5 mM MgSO_4_. DNA was pretreated for up to 3 hrs at 37°C in order to neutralize the antimicrobial activity. To determine if DNA killing required direct cell contact, 2% w/v fish sperm DNA (USB) was resuspended in 10 mM Tris pH 7.4 was placed in sealed dialysis membranes (MW cutoff 3500 Da) and allowed to dialyze into 10 mM Tris pH 7.4 for 4 hours, exchanging the buffer every hour. Cells from mid-log *P. aeruginosa* PAO1 cultures (1 × 10^7^ CFU) were washed into 10 mM Tris pH 7.4 and coincubated directly with 1% or 0.125% dialyzed DNA (final concentration), with 1% eDNA maintained inside dialysis tubing, or 10 mM Tris pH 7.4 alone as a negative control. For histone killing experiments, 1 × 10^7^ CFU mid-log growth phase *P. aeruginosa* PAO1, *E. coli* and *S. aureus* were washed into 10 mM Tris pH 7.4 and subsequently coincubated directly with 1.5 µg/mL calf thymus histones (Roche). Killing experiments were performed at RT in 96-well microplates and bacterial survival was assessed by colony counts (CFU/ml) every hour. All survival values shown are the mean from 4–8 individual replicates and each experiment was performed at least 3 times. Differences in bacterial survival were statistically analyzed by two-tailed student t-test.

### Outer membrane damage visualization

PAO1::OM-lipoChFP was used as an indicator for outer membrane damage. This strain of PAO1 expresses a synthetic Cherry fluorescent lipoprotein (CSFP^OmlA^-ChFP) anchored to the outer membrane encoded on plasmid pCHAP6656 [38]. PAO1::OM-lipoChFP was exposed to a lethal concentration of 2% w/v (20 mg/ml) extracellular sperm DNA (USB) or 2 mM EDTA and red fluorescence of untreated and DNA killed cells were monitored as described above. Fluorescent outer membrane vesicles (OMVs) were counted in 6 fields of view by ImageJ quantification using a manually controlled threshold cutoff.

### DNA, histone, and CAP-mediated induction of *PA4774::lux*

Overnight cultures were grown in LB medium, diluted 1/100 (approximately 1 × 10^7^ CFU) into 100 µl of HBSS medium lacking cations (Life Technologies) in 96-well black plates with a transparent bottom (Thermo Scientific) and overlaid with 75 µl of mineral oil (Sigma Aldrich) to prevent evaporation. Microplate planktonic cultures were incubated at 37°C in a Wallac Victor^3^ luminescence plate reader (Perkin-Elmer) and optical density (growth, OD_600_) and luminescence (gene expression, CPS) readings were taken every 20 minutes in the presence of 0.2% salmon sperm DNA, 0.125 µg/mL polymyxin B and colistin, and 0.1 µg/mL calf thymus histones (Roche). Mean gene expression was derived from triplicate samples at 180 minutes after initial dilution and error bars represent the standard deviation from 4 individual replicates. Differences were statistically assessed by two-tailed student t-test.

### Deoxyribonuclease assay

Overnight cultures of *S. aureus* and *E. coli* were grown in BHI medium and *P. aeruginosa* in BM2 medium, normalized to an OD_600_ = 1 and supernatants were collected by centrifugation at 8000 rpm for 3 minutes. 15 µL of supernatant was incubated with 5 µg of *P. aeruginosa* genomic DNA for 1 h at 37°C. *Pseudomonas aeruginosa* genomic DNA was purified using the Wizard Genomic DNA purification kit (Promega). DNA degradation was visualized on red safe (FroggaBio) stained 1% agarose gels. To test whether exposure to NETs induced DNase production, supernatants from *S. aureus, E. coli* and *P. aeruginosa* incubated in HBSS lacking cations with 10^6^ PMA-stimulated human neutrophils (MOI 10:1, same method as described in the NET killing experiments section) were collected by centrifugation at 8000 rpm for 3 minutes. 100 µL of the supernatants were then coincubated at 37°C with 5 µg salmon sperm DNA stained with 2.5 µM Sytox green. 90 kU of DNase I was included as a positive control. Reactions were placed in 96-well black plates with a transparent bottom and Sytox green fluorescence quantified after 1 hour in a Wallac Victor^3^ luminescence plate reader.

### Neutrophil elastase assay

To determine whether phosphatase treatment or the presence of excess Mg^2+^ altered NET-bound protein function, 2 × 10^5^ human neutrophils were seeded in 96-well black plates with a transparent bottom and induced with 100 nM PMA. Immediately after PMA addition, 50 units of phosphatase (CIAP) and 5 mM MgSO_4_ were added to wells. The plate was then placed in cell culture conditions for 2 hours (37°C, 5% CO_2_). 300 µM elastase substrate I was added to all wells (Calbiochem), which were subsequently overlaid with 75 µL of mineral oil. The plate was then placed in a Wallac Victor^3^ luminescence plate reader at 37°C. Neutrophil elastase activity was monitored by measuring absorbance at OD_410_ nm every 20 minutes for 8 hours.

### Statistical analysis

Statistical analysis was performed using GraphPad Prism v4.0 software. One-way ANOVA with Bonferroni posts tests and two-tailed students t-tests were used to calculate significant differences for plate counts, luminescence and flow cytometry analyses. Significant differences refer to P< 0.05 or less, or as otherwise denoted.

### Ethics statement

Human neutrophils were isolated from human blood samples with ethical approval by the University of Calgary Research Ethics Committee (Ethics ID# 23187), where all subjects provided written informed consent. All animal protocols were approved by the animal care committee of the University of Calgary under the protocol number AC12–0222. All protocols used were in accordance with the guidelines drafted by the University of Calgary Animal Care Committee and the Canadian Council on the Use of Laboratory Animals.

## Supporting Information

S1 Movie Live neutrophils undergo phagocytosis of *Pseudomonas aeruginosa* during *in vivo* skin infection. Spinning-disk confocal intravital microscopy was performed using anti-GR1 (blue) to visualize neutrophils and ChFP-labelled *P. aeruginosa*.
(MOV)

S1 Table Strains, plasmids and primers used in this study. (DOCX)

S1 Fig *Pseudomonas* resistance to NETs is not due to overproduction of secreted DNase. **(A)** DNase activity of 10^7^ CFU *P. aeruginosa*, *S. aureus*, and *E. coli* supernatants isolated from the coincubation with PMA-stimulated human neutrophils in HBSS lacking cations after early (0 hour) and late (4 hour) time points. DNase activity was monitored by loss of Sytox green fluorescence of 5 µg salmon sperm DNA as measured by plate-based spectrophotometer. Relative DNase activity was derived by comparing the Sytox fluorescence of salmon sperm DNA in the presence of bacterial supernatants versus to DNA alone. Degradation assays were incubated at 37°C for 1 hour and were carried out in triplicate. 90 kU/mL of DNase as positive control. **(B)** DNase activity of *P. aeruginosa*, *S. aureus*, and *E. coli* supernatants derived from saturated stationary-phase cultures. Assays were carried out with supernatants from cultures grown in BM2 (*P. aeruginosa*) or BHI (*S. aureus* and *E. coli*). Cell-free supernatants were coincubated 5 µg PAO1 genomic DNA with and without the addition of 10 mM each Ca^2+^ and Mg^2+^ cations.
(TIF)

S2 Fig PMA-induced NETosis exhibits donor-to-donor variation. Human neutrophils were stimulated with PMA or left untreated and NETosis was measured by Sytox green fluorescence. PMA-induced NETosis displays donor-to-donor variation in the kinetics and amplitude of extracellular DNA release as quantified by 2.5 µM Sytox green staining. The presence or absence of 100 µg/ml cytochalasin D had no effect on PMA-induced NETosis. After stimulation, 10^4^ neutrophils were incubated under cell culture conditions (37°C, 5% CO_2_) and NETosis quantified by plate-based fluorescence spectrometer every two hours. Shown are the means of six replicates from each donor.
(TIF)

S3 Fig Cation chelation by EDTA induces membrane destabilization. (**A**)Visualization of the outer membrane integrity of *P. aeruginosa* PAO1 expressing an outer membrane-localized mCherry fluorescent (OM-lipoChFP) lipoprotein [38] immediately after 2 mM EDTA exposure. Insets represent increased magnification of presented micrographs. (**B**) Flow cytometry of EDTA-exposed *P. aeruginosa* PAO1 using SYTO9-PI dual staining as a measure of membrane-compromised bacteria [28]. 2.5 × 10^7^ CFU *P. aeruginosa* PAO1 were exposed to 10 µM EDTA alone or 10 mM Tris pH 7.4 then immediately analyzed by the collection of positive events (N = 50 000) by BD LSRII. Numbers in corners represent the % of 50 000 events that fall into each quadrant gate.
(TIF)

S4 Fig Antibacterial effect exerted by DNA requires direct contact. *P. aeruginosa* PAO1 killing assay in the presence of dialyzed salmon sperm DNA. Dialyzed DNA was either directly added to 1 × 10^7^ CFU PAO1 or separated by dialysis tubing (sep.) (MW cutoff 3,500) and bacterial viability assessed by colony count. Statistically significant differences in bacterial survival relative to the initial bacterial titre is indicated by ***; 2-tailed student t-test (P<0.01). Error bars represent standard deviation. Experiments were repeated three times and the data from one representative experiment is presented.
(TIF)

S5 Fig Neutrophil elastase remains active in PTase- and Mg^2+^-treated NETs. Time course analysis of neutrophil elastase (NE) activity in unstimulated or PMA-stimulated neutrophils in the presence of 50U of phosphatase (PTase) and excess 5 mM Mg^2+^ cations. NE activity was quantified by monitoring cleavage of 300 µM elastase substrate I as measured by absorbance at 410 nm every 20 minutes in a plate-based spectrophotometer over 8 hours at 37°C.
(TIF)

S6 Fig Induction of protective surface modification genes by neutrophil extracellular traps is blocked by treatments targeting DNA. Reporter gene expression from (**A**) spermidine synthesis gene *PA4774::lux* or (**B**) the aminoarabinose LPS modification gene *PA3553::lux* was monitored during coincubation with PMA-activated neutrophils. After 4 hours, the total luminescence (CPS) was measured as an indicator of gene expression. To attempt to prevent NET induction of these operons, exogenous DNase, PTase or Mg^2+^ was added to the coincubation. Values shown are the means and standard error from triplicate replicates. **P<0.01, ***P<0.001 versus no NET exposure (white bar); #P<0.05, ##P<0.01, ###P<0.001 versus DNA exposure (black bar).
(TIF)

S7 Fig NET components differentially induce the expression of the protective spermidine surface modification. Effects of 0.2% salmon sperm DNA, 0.1 µg/mL histone, 0.125 µg/mL polymyxin B and 0.125 µg/mL colistin on the expression of the *PA4774::lux* transcriptional fusion in planktonic cultures. Gene expression was normalized to growth in HBSS buffer after 180 minutes for each condition and CPS/OD600 values are presented. Statistically significant differences (asterisk) in gene induction were determined by 2-tailed student t-tests. * P< 0.05; **P<0.01; ***P<0.0001 between HBSS and DNA/peptide exposure. Expression analysis was performed at least three times and representative means and standard deviations derived from three replicates are shown.
(TIF)
