# Supplementary material for: DNA Is an Antimicrobial Component of Neutrophil Extracellular Traps
Source: PLoS Pathog. 2015 Jan 15;11(1):e1004593. doi: 10.1371/journal.ppat.1004593 (PMC4295883; doi:10.1371/journal.ppat.1004593)
Supplement: S1 Table — (DOCX) [file ppat.1004593.s002.docx]

**Supplemental Table S1.** Strains, plasmids and primers used in this study.

| **Strain** | **Description** | **Reference** |
| --- | --- | --- |
| PAO1 | Wild type *P. aeruginosa* | RE Hancock |
| PAO1::p*16Slux* | Wild type *P. aeruginosa* with transcriptional *luxCDABE* fusion of 16S rRNA genes | [1] |
| PAO1 Tn*7::gfp* | Wild type *P. aeruginosa* constitutively producing Gfp | [2] |
| *S. aureus* | Wild type *S. aureus* ATCC 25923 | [3] |
| *E. coli* | Wild type *E. coli* DH5α | Invitrogen |
| *PA4773/4::lux* | Intergenic *PA4773/4774::lux* transposon mutant and transcriptional fusion, 11_A9 | [4] |
| *PA4773::lux* | *PA4773::lux* transposon mutant and transcriptional fusion, 50_F5 | [4] |
| *PA4774:*:*lux* | *PA4774::lux* transposon mutant and transcriptional fusion, 38_F9 | [4] |
| *PA4774c.c* | *PA4774::lux* chromosomally complemented with *PA4773-PA4775* integrated into *att*Tn*7* site | [5] |
| *PA3553::lux* | *PA3553::lux* transposon mutant and transcriptional fusion, 53_D10 | [4] |
| *PA3559::lux* | *PA3559::lux* transposon mutant and transcriptional fusion, 54_D3 | [4] |
| pσ70-*lux* | Kan^r^, low copy expression vector with σ70 promoter-*luxCDABE* fusion in pSC101 | [6] |
| pCHAP6656 | ChFP with lipoprotein signal peptide, outer membrane anchored | [7] |
| pUCP3559 | PA3559 cloned as a BamHI-HindIII fragment into pUCP22. | This study |
| PA3559For | CCGGATCCGAGGAAAGCACCATGAAGATC |  |
| PA3559Rev | CCAAGCTTTCAGGCCTGGCGCAACAGGCG |  |

**References**

1. Riedel CU, Casey PG, Mulcahy H, O'Gara F, Gahan CG, et al. (2007) Construction of p16Slux, a novel vector for improved bioluminescent labeling of Gram-negative bacteria. Appl Environ Microbiol 73(21): 7092-7095.

2. Koch B, Jensen LE, Nybroe O. (2001) A panel of Tn*7*-based vectors for insertion of the *gfp* marker gene or for delivery of cloned DNA into Gram-negative bacteria at a neutral chromosomal site. J Microbiol Methods 45(3): 187-195.

3. Pilsczek FH, Salina D, Poon KK, Fahey C, Yipp BG, et al. (2010) A novel mechanism of rapid nuclear neutrophil extracellular trap formation in response to *Staphylococcus aureus*. J Immunol 185(12): 7413-7425.

4. Lewenza S, Falsafi RK, Winsor G, Gooderham WJ, McPhee JB, et al. (2005) Construction of a mini-Tn5-luxCDABE mutant library in *Pseudomonas aeruginosa* PAO1: A tool for identifying differentially regulated genes. Genome Res 15(4): 583-589.

5. Johnson L, Mulcahy H, Kanevets U, Shi Y, Lewenza S. (2012) Surface-localized spermidine protects the *Pseudomonas aeruginosa* outer membrane from antibiotic treatment and oxidative stress. J Bacteriol 194(4): 813-826.

6. Davidson CJ, Narang A, Surette MG. (2010) Integration of transcriptional inputs at promoters of the arabinose catabolic pathway. BMC Syst Biol 4: 75-0509-4-75.

7. Lewenza S, Mhlanga MM, Pugsley AP. (2008) Novel inner membrane retention signals in *Pseudomonas aeruginosa* lipoproteins. J Bacteriol 190(18):6119-6125.
