# Supplementary figures and images for: DNA Is an Antimicrobial Component of Neutrophil Extracellular Traps
Source: PLoS Pathog. 2015 Jan 15;11(1):e1004593. doi: 10.1371/journal.ppat.1004593 (PMC4295883; doi:10.1371/journal.ppat.1004593)

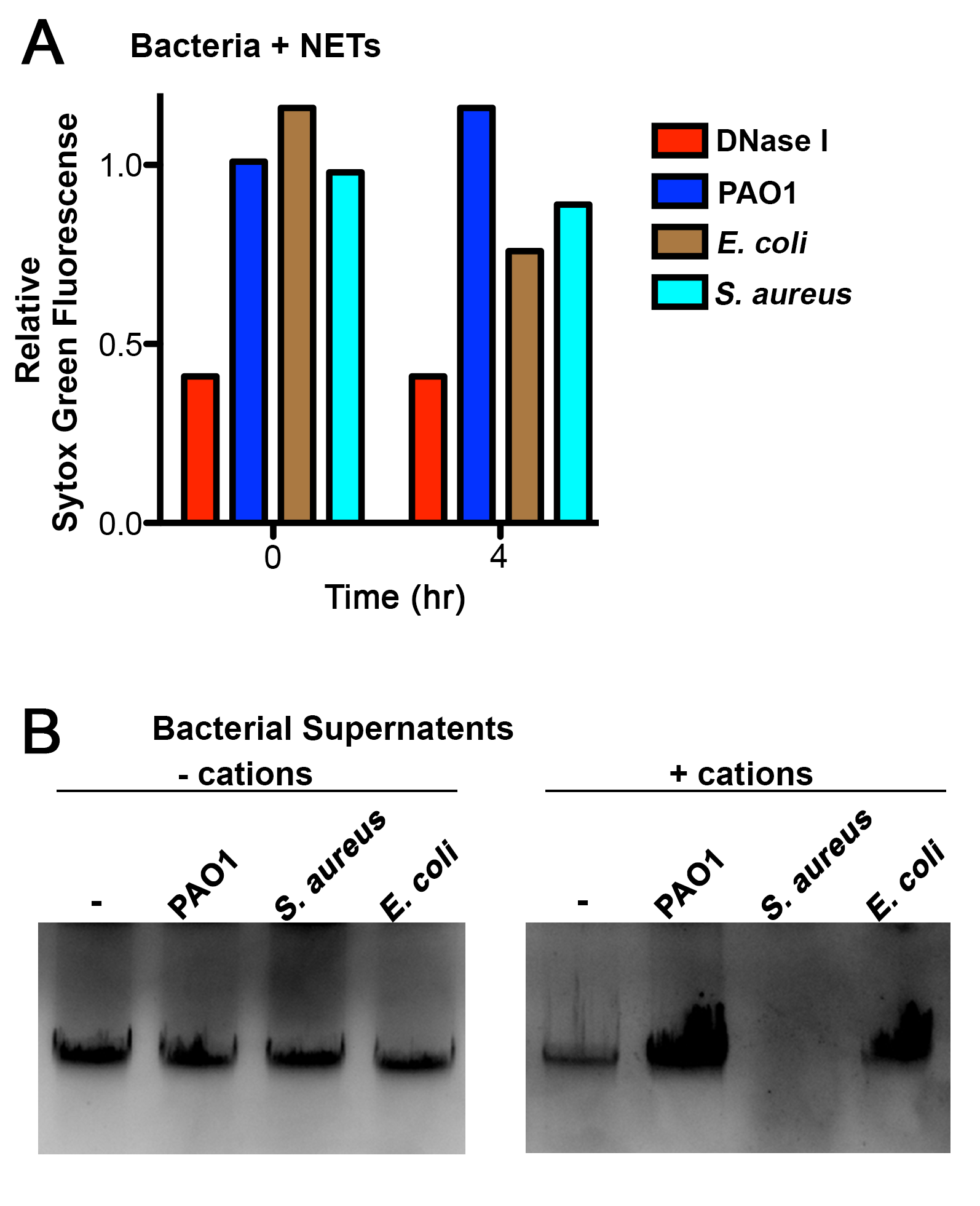

Supplement: S1 Fig — (A) DNase activity of 107 CFU P. aeruginosa, S. aureus, and E. coli supernatants isolated from the coincubation with PMA-stimulated human neutrophils in HBSS lacking cations after early (0 hour) and late (4 hour) time points. DNase activity was monitored by loss of Sytox green fluorescence of 5 µg salmon sperm DNA as measured by plate-based spectrophotometer. Relative DNase activity was derived by comparing the Sytox fluorescence of salmon sperm DNA in the presence of bacterial supernatants versus to DNA alone. Degradation assays were incubated at 37°C for 1 hour and were carried out in triplicate. 90 kU/mL of DNase as positive control. (B) DNase activity of P. aeruginosa, S. aureus, and E. coli supernatants derived from saturated stationary-phase cultures. Assays were carried out with supernatants from cultures grown in BM2 (P. aeruginosa) or BHI (S. aureus and E. coli). Cell-free supernatants were coincubated 5 µg PAO1 genomic DNA with and without the addition of 10 mM each Ca2+ and Mg2+ cations. (TIF) [file ppat.1004593.s003.tif]

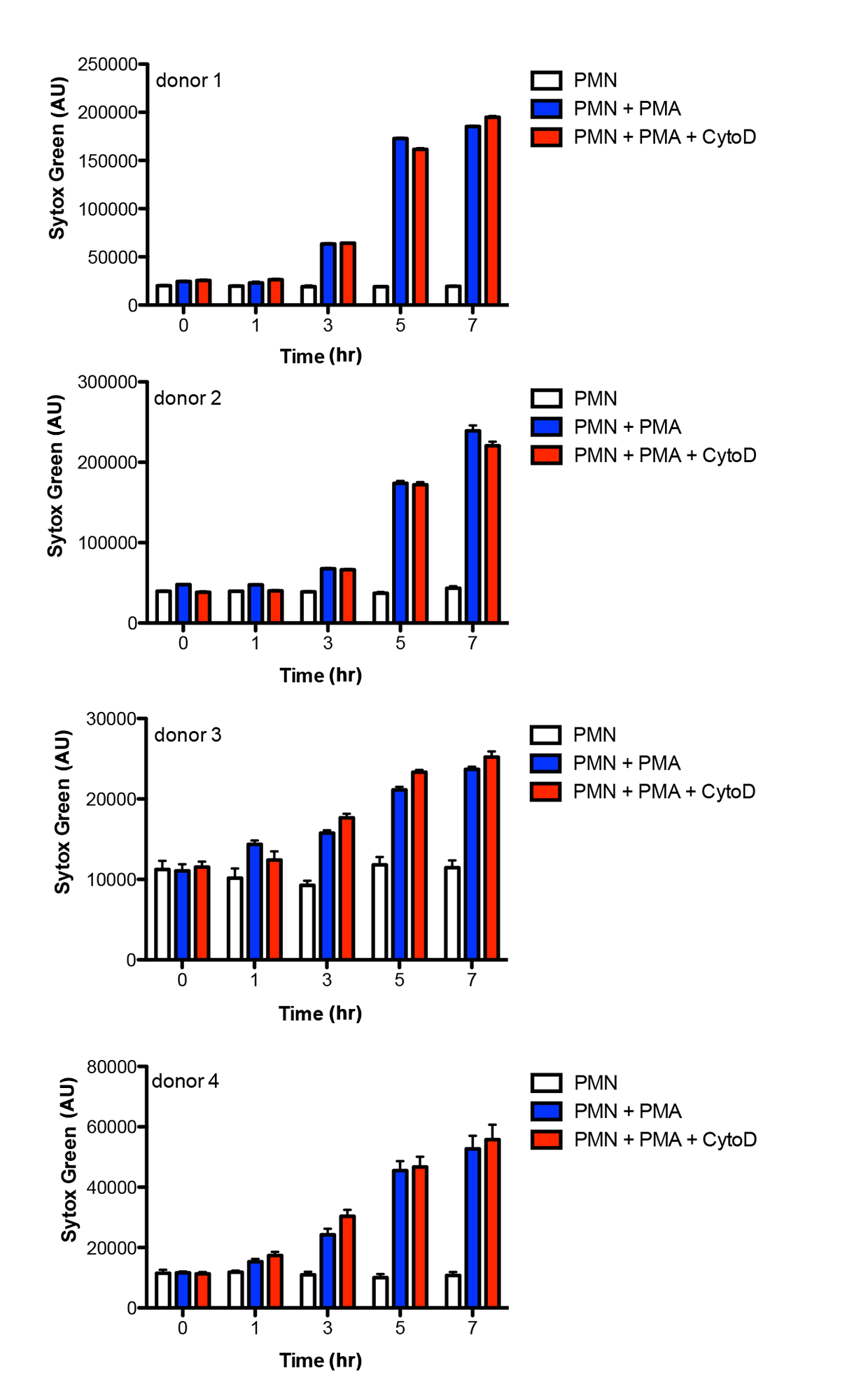

Supplement: S2 Fig — Human neutrophils were stimulated with PMA or left untreated and NETosis was measured by Sytox green fluorescence. PMA-induced NETosis displays donor-to-donor variation in the kinetics and amplitude of extracellular DNA release as quantified by 2.5 µM Sytox green staining. The presence or absence of 100 µg/ml cytochalasin D had no effect on PMA-induced NETosis. After stimulation, 104 neutrophils were incubated under cell culture conditions (37°C, 5% CO2) and NETosis quantified by plate-based fluorescence spectrometer every two hours. Shown are the means of six replicates from each donor. (TIF) [file ppat.1004593.s004.tif]

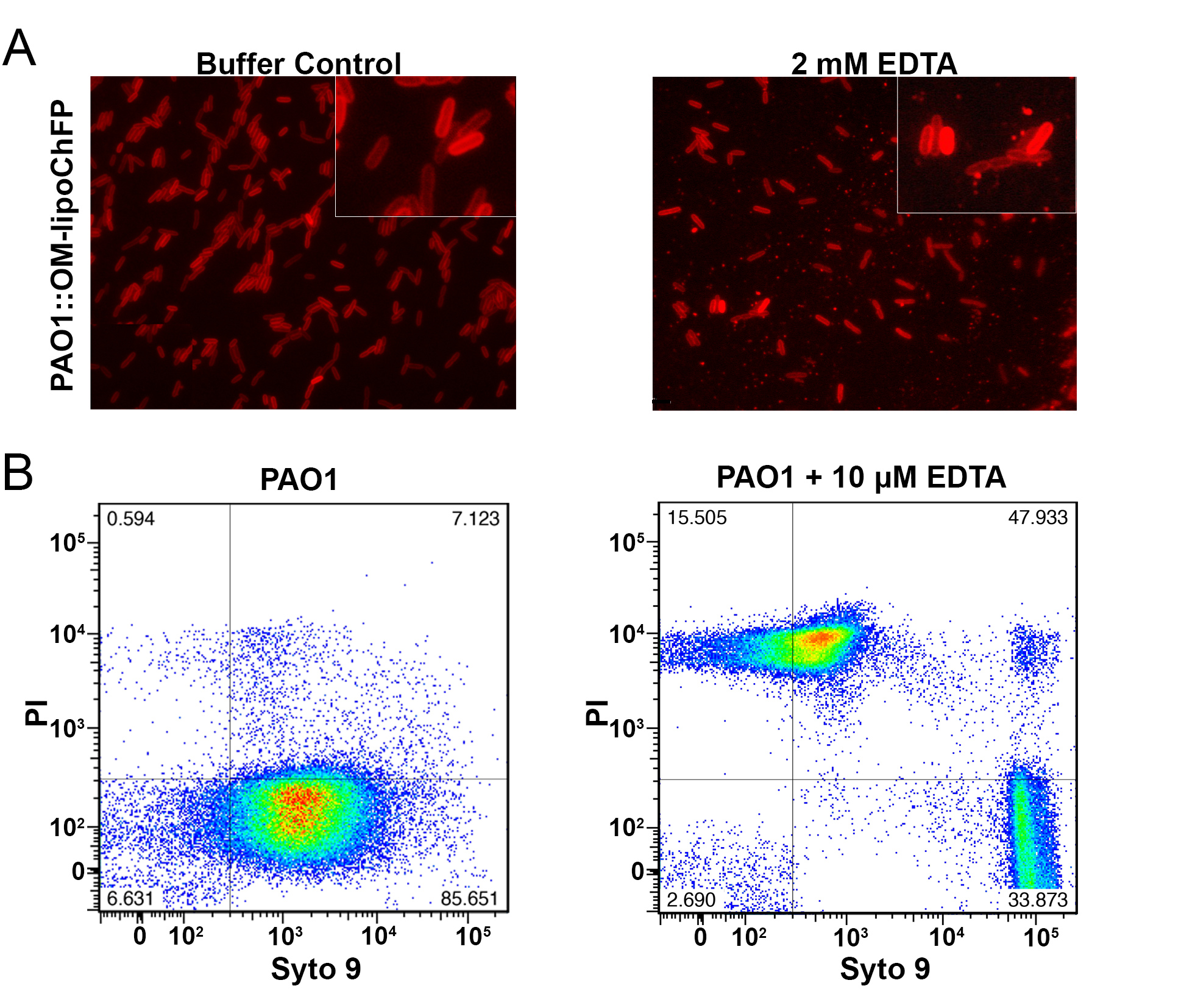

Supplement: S3 Fig — (A)Visualization of the outer membrane integrity of P. aeruginosa PAO1 expressing an outer membrane-localized mCherry fluorescent (OM-lipoChFP) lipoprotein [38] immediately after 2 mM EDTA exposure. Insets represent increased magnification of presented micrographs. (B) Flow cytometry of EDTA-exposed P. aeruginosa PAO1 using SYTO9-PI dual staining as a measure of membrane-compromised bacteria [28]. 2.5 × 10^7^ CFU P. aeruginosa PAO1 were exposed to 10 µM EDTA alone or 10 mM Tris pH 7.4 then immediately analyzed by the collection of positive events (N = 50 000) by BD LSRII. Numbers in corners represent the % of 50 000 events that fall into each quadrant gate. (TIF) [file ppat.1004593.s005.tif]

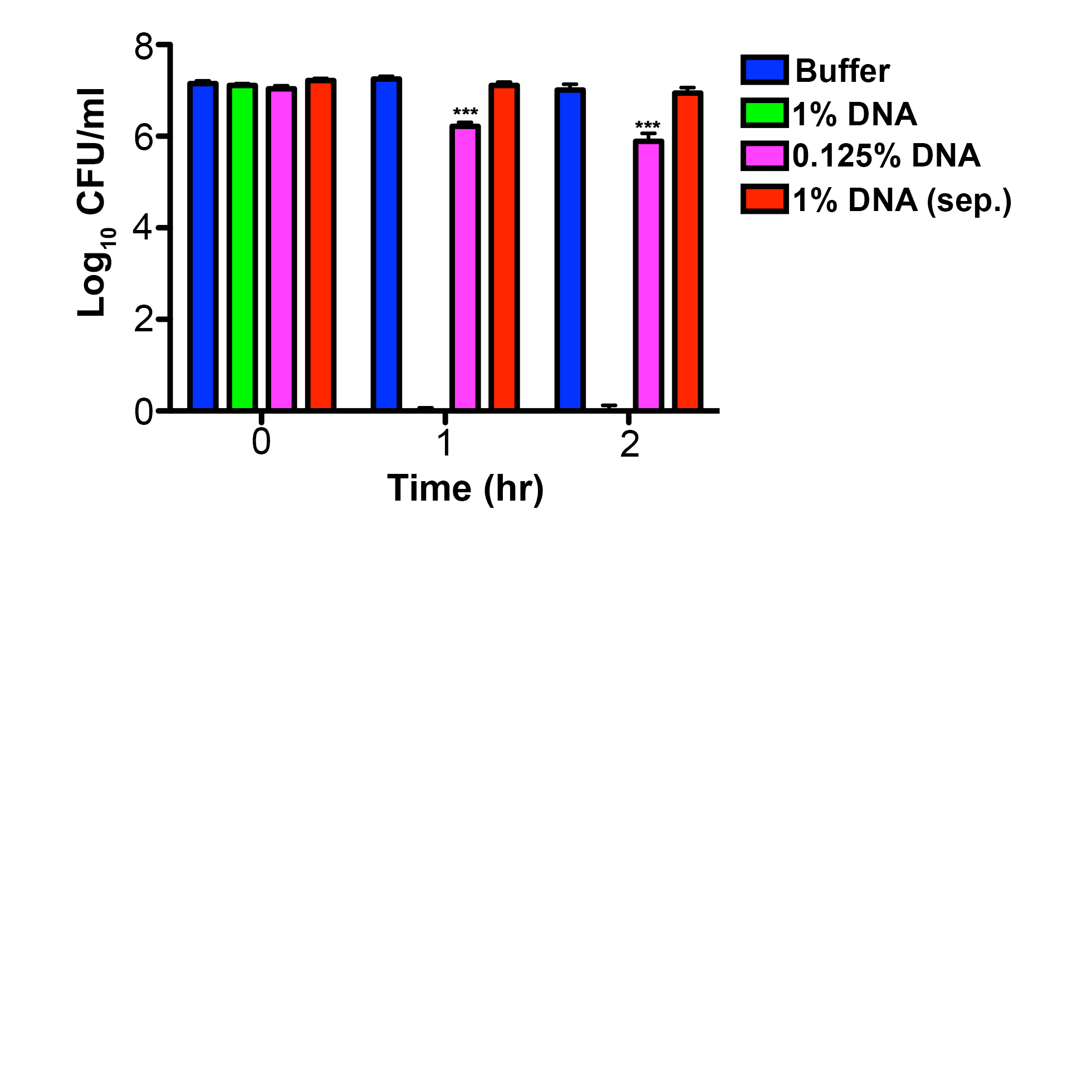

Supplement: S4 Fig — P. aeruginosa PAO1 killing assay in the presence of dialyzed salmon sperm DNA. Dialyzed DNA was either directly added to 1 × 10^7^ CFU PAO1 or separated by dialysis tubing (sep.) (MW cutoff 3,500) and bacterial viability assessed by colony count. Statistically significant differences in bacterial survival relative to the initial bacterial titre is indicated by ***; 2-tailed student t-test (P<0.01). Error bars represent standard deviation. Experiments were repeated three times and the data from one representative experiment is presented. (TIF) [file ppat.1004593.s006.tif]

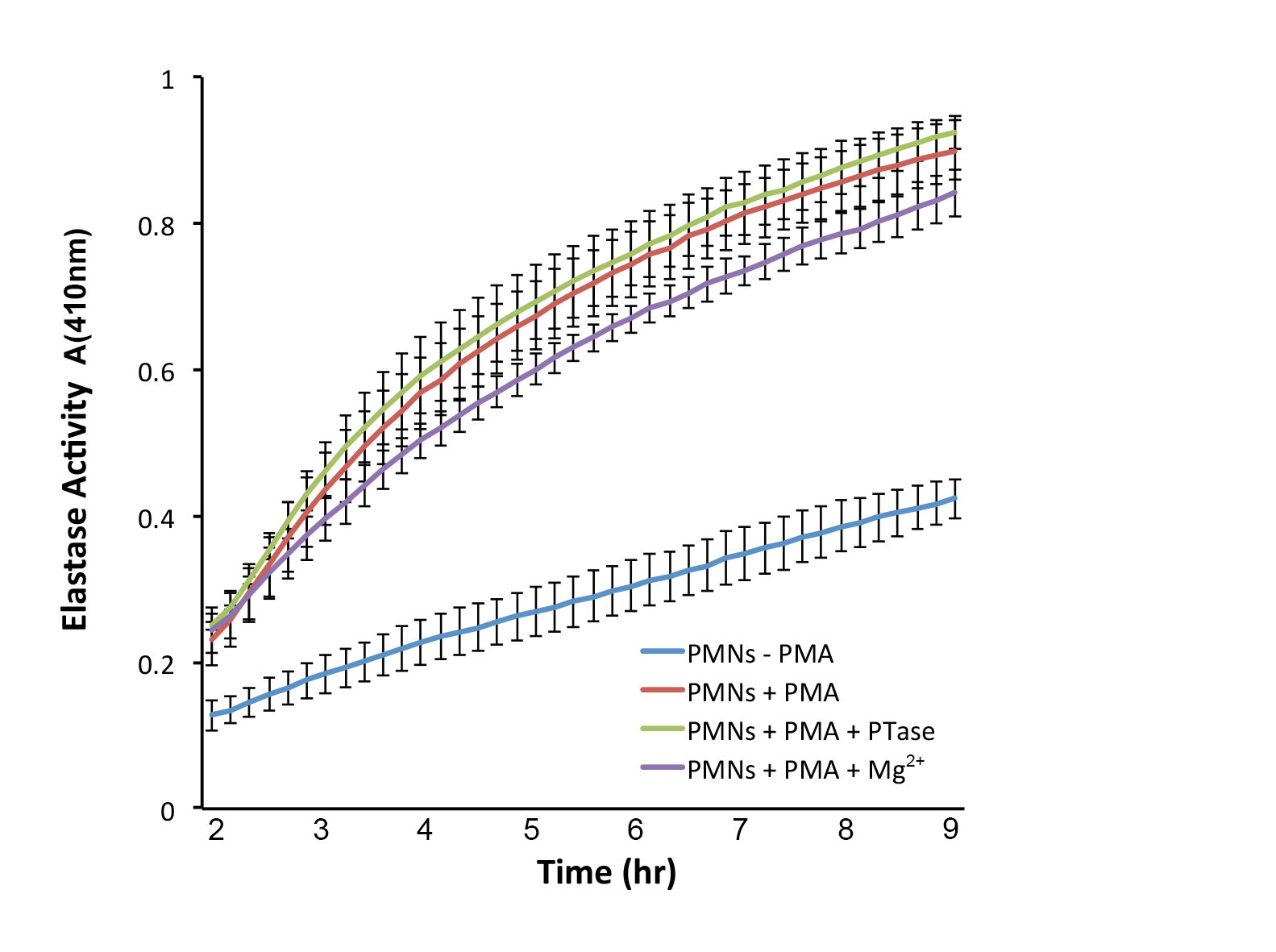

Supplement: S5 Fig — Time course analysis of neutrophil elastase (NE) activity in unstimulated or PMA-stimulated neutrophils in the presence of 50U of phosphatase (PTase) and excess 5 mM Mg2+ cations. NE activity was quantified by monitoring cleavage of 300 µM elastase substrate I as measured by absorbance at 410 nm every 20 minutes in a plate-based spectrophotometer over 8 hours at 37°C. (TIF) [file ppat.1004593.s007.tif]

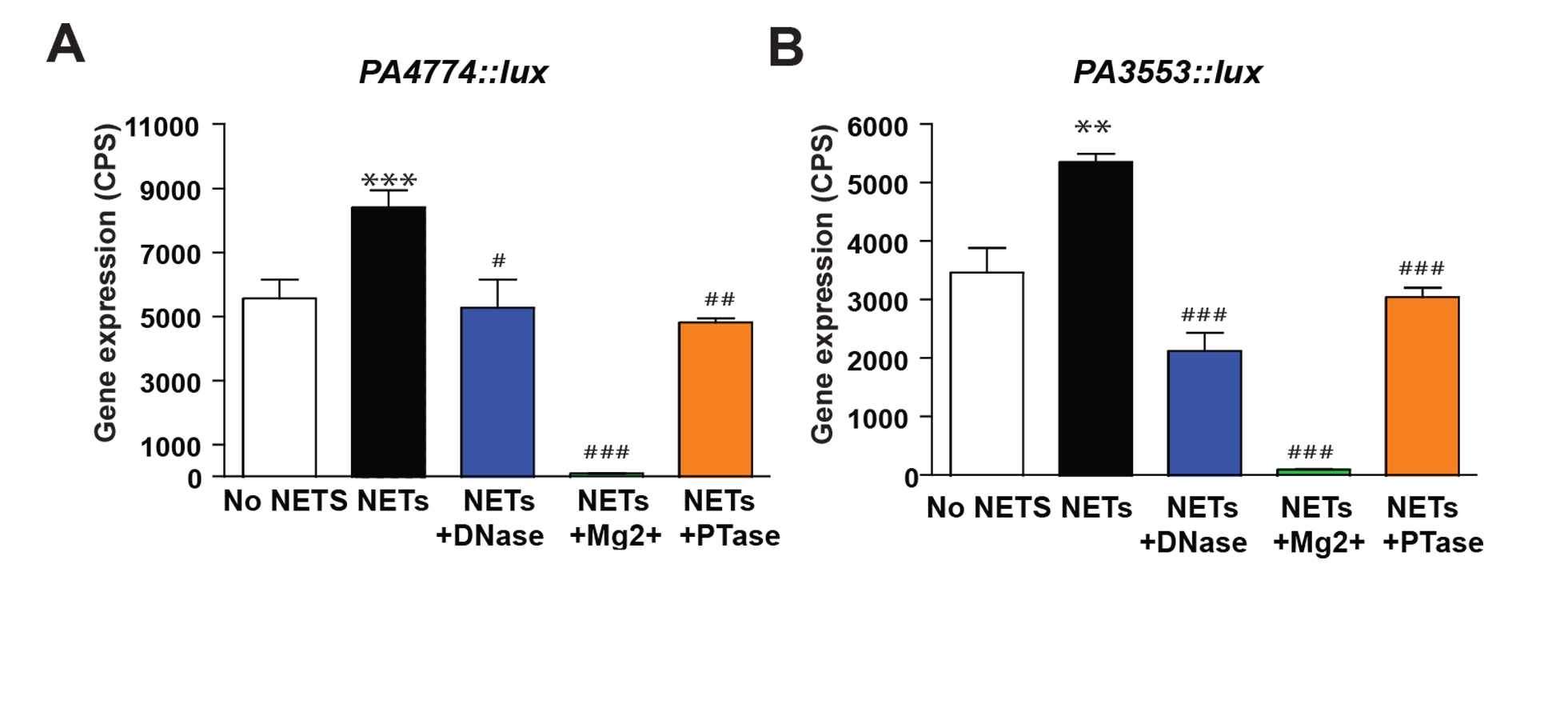

Supplement: S6 Fig — Reporter gene expression from (A) spermidine synthesis gene PA4774::lux or (B) the aminoarabinose LPS modification gene PA3553::lux was monitored during coincubation with PMA-activated neutrophils. After 4 hours, the total luminescence (CPS) was measured as an indicator of gene expression. To attempt to prevent NET induction of these operons, exogenous DNase, PTase or Mg2+ was added to the coincubation. Values shown are the means and standard error from triplicate replicates. **P<0.01, ***P<0.001 versus no NET exposure (white bar); #P<0.05, ##P<0.01, ###P<0.001 versus DNA exposure (black bar). (TIF) [file ppat.1004593.s008.tif]

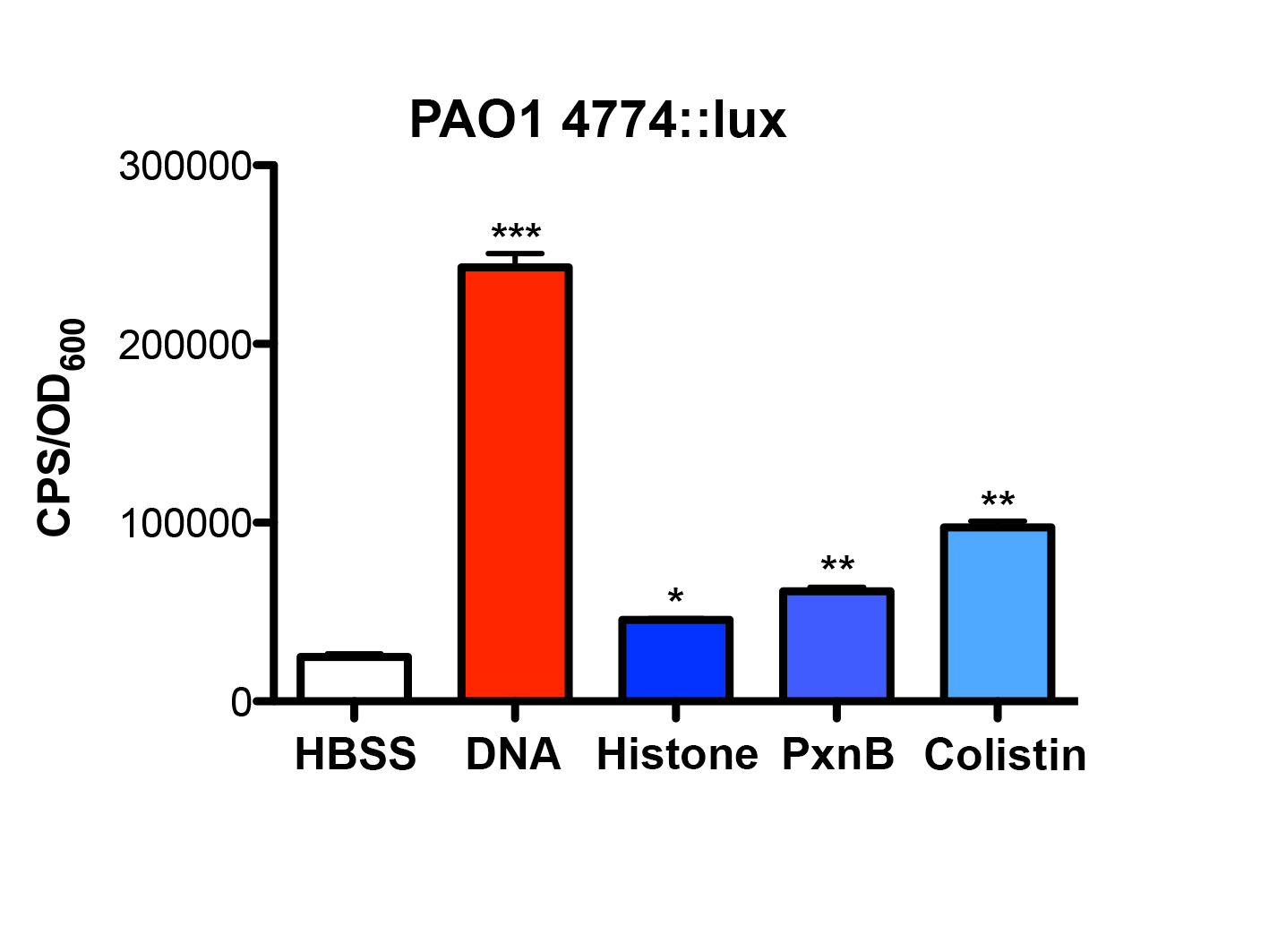

Supplement: S7 Fig — Effects of 0.2% salmon sperm DNA, 0.1 µg/mL histone, 0.125 µg/mL polymyxin B and 0.125 µg/mL colistin on the expression of the PA4774::lux transcriptional fusion in planktonic cultures. Gene expression was normalized to growth in HBSS buffer after 180 minutes for each condition and CPS/OD600 values are presented. Statistically significant differences (asterisk) in gene induction were determined by 2-tailed student t-tests. * P< 0.05; **P<0.01; ***P<0.0001 between HBSS and DNA/peptide exposure. Expression analysis was performed at least three times and representative means and standard deviations derived from three replicates are shown. (TIF) [file ppat.1004593.s009.tif]
